# Origin of Saxitoxin Biosynthetic Genes in Cyanobacteria

**DOI:** 10.1371/journal.pone.0005758

**Published:** 2009-06-01

**Authors:** Ahmed Moustafa, Jeannette E. Loram, Jeremiah D. Hackett, Donald M. Anderson, F. Gerald Plumley, Debashish Bhattacharya

**Affiliations:** 1 Interdisciplinary Program in Genetics, University of Iowa, Iowa City, Iowa, United States of America; 2 Bermuda Institute of Ocean Sciences, St. George's, Bermuda; 3 Ecology and Evolutionary Biology Department, University of Arizona, Tucson, Arizona, United States of America; 4 Woods Hole Oceanographic Institution, Woods Hole, Massachusetts, United States of America; 5 Department of Biology, University of Iowa, Iowa City, Iowa, United States of America; University of California, Berkeley, United States of America

## Abstract

**Background:**

Paralytic shellfish poisoning (PSP) is a potentially fatal syndrome associated with the consumption of shellfish that have accumulated saxitoxin (STX). STX is produced by microscopic marine dinoflagellate algae. Little is known about the origin and spread of saxitoxin genes in these under-studied eukaryotes. Fortuitously, some freshwater cyanobacteria also produce STX, providing an ideal model for studying its biosynthesis. Here we focus on saxitoxin-producing cyanobacteria and their non-toxic sisters to elucidate the origin of genes involved in the putative STX biosynthetic pathway.

**Methodology/Principal Findings:**

We generated a draft genome assembly of the saxitoxin-producing (STX+) cyanobacterium *Anabaena circinalis* ACBU02 and searched for 26 candidate saxitoxin­genes (named *sxtA* to *sxtZ*) that were recently identified in the toxic strain *Cylindrospermopsis raciborskii* T3. We also generated a draft assembly of the non-toxic (STX−) sister *Anabaena circinalis* ACFR02 to aid the identification of saxitoxin-specific genes. Comparative phylogenomic analyses revealed that nine putative STX genes were horizontally transferred from non-cyanobacterial sources, whereas one key gene (*sxtA*) originated in STX+ cyanobacteria *via* two independent horizontal transfers followed by fusion. In total, of the 26 candidate saxitoxin-genes, 13 are of cyanobacterial provenance and are monophyletic among the STX+ taxa, four are shared amongst STX+ and STX-cyanobacteria, and the remaining nine genes are specific to STX+ cyanobacteria.

**Conclusions/Significance:**

Our results provide evidence that the assembly of STX genes in ACBU02 involved multiple HGT events from different sources followed presumably by coordination of the expression of foreign and native genes in the common ancestor of STX+ cyanobacteria. The ability to produce saxitoxin was subsequently lost multiple independent times resulting in a nested relationship of STX+ and STX− strains among *Anabaena circinalis* strains.

## Introduction

Paralytic shellfish poisoning (PSP) is a potentially fatal syndrome associated with the consumption of shellfish that have accumulated toxins produced by microscopic marine algae. This phenomenon is the most widespread of the poisoning syndromes caused by blooms of toxic algae, commonly referred to as “red tides” or “harmful algal blooms”, (HABs). The impacts of HABs on marine ecosystems and the seafood industry are substantial. STX, the etiological agent of PSP, is produced by a small number of marine dinoflagellates and freshwater filamentous cyanobacteria [1]. These latter taxa are a promising model for identifying putative saxitoxin genes and to elucidating their evolutionary history because of the small genome sizes of cyanobacteria and the wealth of available prokaryotic genomic data. Knowledge about STX genes and their regulation in cyanobacteria could potentially help in the future ameliorate the most significant impacts from STX toxicity that derive from dinoflagellate red tides in marine systems.

Recent analyses of STX-producing cyanobacteria have significantly advanced our understanding of saxitoxin biosynthesis *via* isolation and characterization of enzymes putatively involved in the STX pathway; e.g., S-adenosylhomocysteine hydrolase, methionine aminopeptidase [2], sulfotransferase [3], Na(+)-dependent transporter [4], aminotransferase, and *O*-carbamoyltransferase [5]. A recent study provided the complete sequence (∼35 kb) of a putative STX biosynthesis gene cluster encoding 26 proteins in the toxic (STX+) strain *Cylindrospermopsis raciborskii* T3. The cluster (with genes named *sxtA* to *sxtZ*) was identified using genome-walking upstream and downstream of the gene encoding *O*-carbamoyltransferase, which was initially isolated using degenerate PCR [6]. Preliminary sequence similarity analyses predicted the putative functions and origins for each of the 26 STX genes. Although not yet substantiated by biochemical or molecular genetic analyses these promising data imply an important role for horizontal gene transfer (HGT) in the assembly of the STX gene cluster in *Cylindrospermopsis raciborskii* T3.

Given this information, here we utilized a combined genomic and phylogenetic approach to investigate in detail the evolutionary history and genomic characteristics of putative STX genes in saxitoxin-producing cyanobacteria. We used the predicted STX gene cluster in *Cylindrospermopsis raciborskii* T3 as “bait” to identify homologs in other toxic and non-toxic strains. To this end, we generated novel genome data from the STX+ cyanobacterium *Anabaena circinalis* ACBU02 and posed the following questions: 1) Are all of the *Cylindrospermopsis raciborskii* T3 candidate STX genes present in ACBU02? 2) If not, which genes and functions form the core set that is conserved across different taxa? and, 3) what are the phylogenetic origins of these genes?

## Results and Discussion

### 16S rRNA phylogeny

**Figure 1:**
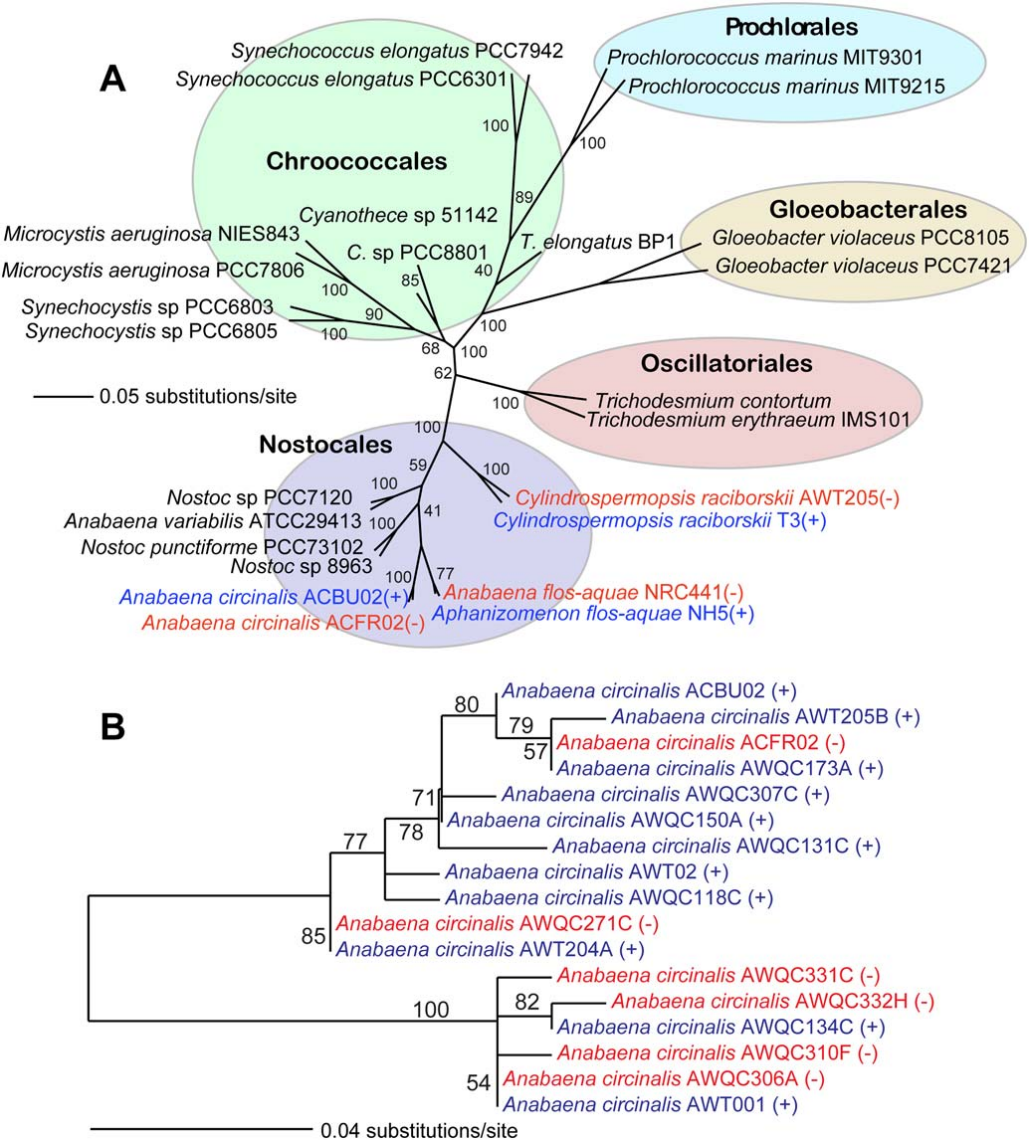
Phylogenetic tree of 16S rRNA from cyanobacteria. Unrooted ML phylogenetic trees inferred from small subunit (16S) rRNA that include (a) different cyanobacterial orders and (b) only saxitoxin-producing (STX+) and STX− strains of *Anabaena circinalis*. ML bootstrap values (when ≥50%) are indicated at the nodes. The branch lengths are proportional to the number of substitutions per site (see scales in the figure). The toxic and non-toxic strains are indicated by the plus sign (+) and minus sign (−), respectively.

We examined the evolutionary relationships between STX+ and STX− cyanobacterial strains by constructing a 16S rRNA phylogenetic tree that focused on this branch of the tree of life (see Figure 1A). The phylogeny indicates that STX+ and STX− strains of the cyanobacterial species, *Anabaena circinalis*, *Aphanizomenon flos-aquae*, and *Cylindrospermopsis raciborskii* are evolutionary closely related and each form monophyletic clades. This suggests that toxicity as a character was either gained in the toxic strains through independent HGTs or alternatively, is an ancestral trait for these cyanobacteria and was lost from the non-toxic strains. An argument that favors the latter scenario is the known complexity of the STX biosynthetic pathway and the low likelihood of convergent evolution of this rare trait among these closely related species. Therefore, we propose that toxicity is an ancestral state that was assembled in the common ancestor of the toxic and non-toxic strains we have studied and most likely some core genes involved in the pathway were lost from STX- taxa. A similar scenario was recently proposed to explain the distribution of toxic and non-toxic strains of the cyanobacterium *Planktothrix* regarding the biosynthesis of microcystin, a cyclic heptapeptide cyanotoxin [7]. In an earlier study, Beltran and Neilan [8] suggested that toxic and non-toxic strains of *Anabaena circinalis* would segregate into two distinct clades. Our *Anabaena circinalis*-specific 16S rRNA tree (Figure 1B) is inconsistent with this idea, clearly showing that STX− taxa are nested within STX+ clades, supporting loss of toxicity in the former group. This pattern results in a polyphyletic collection of STX− strains.

### Genome annotation of saxitoxin-producing Anabaena circinalis strain ACBU02

A total of 181 contigs were assembled from the ACBU02 “454” genome data using the Newbler and SeqMan Pro assemblers. These data total 4.5 Mb of unique sequence that agrees well with the pulse-field gel electrophoresis estimate of genome size (4.0–4.5 Mb) in this taxon [9]. We believe therefore that the draft assembly of ACBU02 represents a near-complete genome for downstream analyses. A total of 5,188 putative protein-coding genes were identified in the assembled genome of ACBU02 using RAST [10] and GeneMarkS [11]. These data are publicly available at the RAST repository ([10]; genome ID: 109265.7). Using BLAST searches against the NCBI non-redundant database “nr”, 4,540 proteins (87.5%) had significant matches (*e*-value<1E-9), 231 proteins (4.5%) had matches with an *e*-value between 1E-3 and 1E-9, and 417 proteins (8.0%) did not have matches (i.e., with an *e*-value<1E-3). Of the 4,771 predicted proteins that had significant hits, 4,474 (93.8%) had the closest matches to cyanobacterial taxa, of which the most frequent matching taxa were *Nostoc punctiforme* (1,450 hits), *Nodularia spumigena* (882 hits), *Anabaena variabilis* (752 hits), and *Nostoc* sp. (594 hits). For the remaining 6.2% of the predicted proteins, the best hits were distributed among other bacterial phyla, of which the most frequent were proteobacteria, firmicutes, and planctomycetes, in descending order.

### Gene families

We identified 3,483 gene families, of which 3,024 (86.8%) are single-copy genes and 30 families (0.86%) contain greater than 10 genes per family. The largest gene family has 212 members (4% of the predicted genes) encoding proteins that contain serine/threonine-specific protein kinases and tetratricopeptide repeat (TPR) domains. The second largest family is of size 84 genes (1.6% of the predicted set) encoding ATP-binding cassette (ABC) transporters.

### Broad patterns of STX gene origins

The 26 putative STX genes identified by Kellmann et al. [6] in the toxic cyanobacterium *Cylindrospermopsis raciborskii* T3 were used as a query to retrieve homologs in GenBank. Taxon sampling was maximized with the use of the NCBI nr protein database with the addition of the predicted proteomes from the STX+ and STX− strains of *Anabaena circinalis* ACBU02 and ACFR02, respectively. Our preliminary assembly of the ACFR02 data identified 583 contigs that sum to 4.4 Mb of unique genome sequence, encoding 5,203 predicted proteins. We have used these data as a rough guide to strengthen our inferences about ACBU02 genome evolution. The ACFR02 genome is currently being completed and will be published in the near future.

Each STX gene query resulted in a comprehensive alignment that was submitted to a bootstrapped maximum-likelihood (ML) phylogenetic analysis [12], [13]. Examination of these 26 phylogenies (see Figure S1) led us to identify three broad evolutionary patterns that underlie and explain the evolutionary history of these putative STX genes. The first pattern consists of genes that are common to STX+ and STX− cyanobacteria (Table 1, group I). The second pattern defines genes that have a cyanobacterial origin in the putative common ancestor of only STX+ cyanobacteria (Table 1, group II). The third pattern (Table 1, groups III, IV, V, and VI) includes genes that have originated *via* HGT in the genome of the putative ancestor of STX+ cyanobacteria from a non-cyanobacterial source. The genes in the second (putatively vertically inherited) and third (putatively horizontally transferred) patterns could have been established in the common ancestor of STX+ and STX− cyanobacteria; however, they were subsequently lost differentially from STX− cyanobacteria. The following sections provide a detailed account and our interpretation of these phylogenetic patterns of STX gene origin.

**Table 1:** Genes involved in the biosynthesis of saxitoxin in cyanobacteria, their putative functional annotation, and origin

STX Gene	Accession	Annotation	Origin	Monophyly	Group
*sxtO*	ABI75115	Adenylylsulfate kinase	Cyanobacteria	N	I
*sxtW*	ABI75106	Ferredoxin	Cyanobacteria	N	I
*sxtY*	ABI75117	Phosphate uptake regulator	Cyanobacteria	N	I
*sxtZ*	ABI75118	Two-component sensor histidine kinase	Cyanobacteria	N	I
*sxtD*	ABI75089	Sterole desaturase	Cyanobacteria	Y	II
*sxtF*	ABI75096	Toxic compound extrusion protein	Cyanobacteria	Y	II
*sxtI*	ABI75099	*O*-carbamoyltransferase	Cyanobacteria	Y	II
*sxtJ*	ABI75100	Hypothetical protein	Cyanobacteria	Y	II
*sxtK*	ABI75101	Hypothetical protein	Cyanobacteria	Y	II
*sxtL*	ABI75102	GDSL-lipase	Cyanobacteria	Y	II
*sxtM*	ABI75103	Toxic compound extrusion protein	Cyanobacteria	Y	II
*sxtN*	ABI75104	Sulfotransferase	Cyanobacteria	Y	II
*sxtP*	ABI75114	Saxitoxin-binding protein	Cyanobacteria	Y	II
*sxtR*	ABI75112	Acyl-CoA N-acyltransferase	Cyanobacteria	Y	II
*sxtU*	ABI75108	Short-chain alcohol dehydrogenase	Cyanobacteria	Y	II
*sxtV*	ABI75107	Fumarate reductase	Cyanobacteria	Y	II
*sxtX*	ABI75105	Cephalosporin hydroxylase	Cyanobacteria	Y	II
*sxtB*	ABI75093	Cytidine deaminase	Proteobacteria	Y	III
*sxtE*	ABI75095	Chaperone-like protein	Proteobacteria	Y	III
*sxtG*	ABI75097	Amidinotransferase	Proteobacteria	Y	III
*sxtQ*	ABI75113	Unknown	Proteobacteria	Y	III
*sxtS*	ABI75110	Phytanoyl-CoA dioxygenase	Proteobacteria	Y	III
*sxtC*	ABI75092	Amidohydrolase	Firmicutes	Y	IV
*sxtH*	ABI75098	Phenylpropionate dioxygenase	Unknown	Y	V
*sxtT*	ABI75109	Phenylpropionate dioxygenase	Unknown	Y	V
*sxtA*	ABI75094	Polyketide synthase	Chimeric	Y	VI

The rows are grouped based on the origin of the genes and the monophyly of the STX+ taxa for the respective gene as following: I = Cyanobacteria and not monophyletic, II = Cyanobacteria and monophyletic, III = Proteobacteria, IV = Firmicutes, V = Unknown, VI = Chimeric.

### Genes of cyanobacterial origin

**Figure 2:**
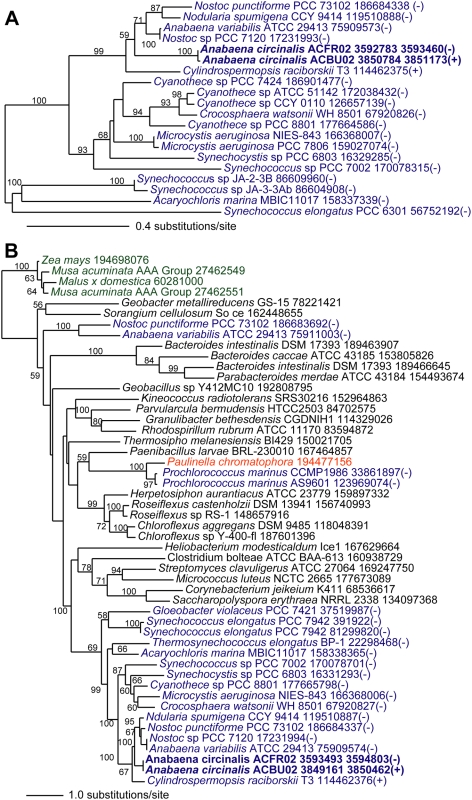
Phylogeny of sxtY and sxtZ. ML phylogenetic trees of (a) sxtY (phosphate uptake regulator) and (b) sxtZ (histidine kinase). These represent the class of saxitoxin-related genes of cyanobacterial origin in which taxa most closely related to the STX+ strains are members of the Nostocaceae. ML bootstrap values (when ≥50%) are indicated at the nodes. The branch lengths are proportional to the number of substitutions per site (see scales in the figure). Cyanobacterial taxa are shown in blue text, non-cyanobacterial prokaryotes in black text, and eukaryotes (Plantae) in green except for the photosynthetic filose amoeba *Paulinella chromatophora* in red. The trees have been rooted arbitrarily.

We find that 17 of the predicted 26 STX genes in *Cylindrospermopsis raciborskii* T3 are of cyanobacterial origin. These genes are unambiguously categorized into two distinct evolutionary groups. One group includes four genes (Table 1, group I) that are broadly distributed across cyanobacteria with their phylogeny agreeing with the typical 16S rRNA tree for these taxa (see Figure 1A). In these cases, there is no phylogenetic distinction between STX+ and STX− species. The phylogeny of sxtY (phosphate uptake regulator) and sxtZ (histidine kinase) exemplify this general topological pattern (Figures 2A and 2B, respectively). Within this group of genes, STX+ and STX− strains of *Anabaena circinalis* are monophyletic (i.e., they share a common ancestor) as expected (i.e., based on the 16S rRNA tree). This clade is positioned within a larger group of cyanobacteria in which the most closely related sequences are members of the Nostocaceae such as the STX− species *Anabaena variabilis*, *Nostoc* sp., and *Nodularia spumigena*. This phylogenetic pattern suggests these genes are not specific to saxitoxin biosynthesis in the STX+ cyanobacteria, but rather have putatively been co-opted for this function. It is worth noting that one gene, sxtW (ferredoxin), did not have a significant hit in the genome of ACBU02, suggesting that it may not provide a core function involved in STX synthesis. Alternatively, the function of sxtW might have been substituted in *Anabaena circinalis* ACBU02 by another gene product.

**Figure 3:**
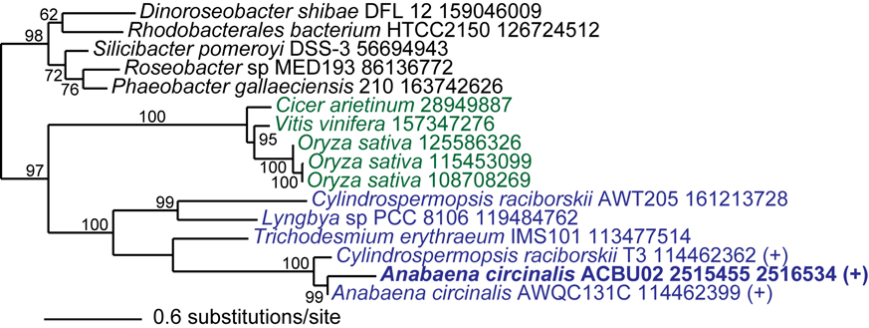
Phylogeny of sxtN. ML phylogenetic tree of sxtN (sulfotransferase) that represents saxitoxin-related genes of cyanobacterial origin that is potentially from members of the Oscillatoriales. ML bootstrap values (when ≥50%) are indicated at the nodes. The branch lengths are proportional to the number of substitutions per site (see scale in the figure). Cyanobacterial taxa are shown in blue text, non-cyanobacterial prokaryotes in black text, and eukaryotes (Plantae) in green. The tree has been rooted on the branch leading to the non-cyanobacterial prokaryotes.

The other STX genes of cyanobacterial origin (13 sequences in 11 gene families) in which the STX-producing taxa form a well-supported monophyletic clade that is positioned within cyanobacteria (Table 1, group II). However, the clade containing STX+ taxa is relatively evolutionarily distant from its closest STX− neighbors (Figure 3). These 12 genes can be further subdivided into four classes based on their closest STX− cyanobacterial neighbor and their distribution among different cyanobacteria. The first comprises three genes that are shared with other Nostocaceae cyanobacteria similar to that of the non-saxitoxin-specific genes identified above, except that STX+ taxa form a clade that excludes STX− taxa. The three genes in this group (Figure S1) are saxitoxin-binding protein (*sxtP*), short-chain alcohol dehydrogenase (*sxtU*), and succinate dehydrogenase/fumarate reductase (*sxtV*). The second class is a set of three genes that are shared with Chroococcales cyanobacteria (e.g., *Synechococcus* sp). This group includes two genes that encode toxic compound extrusion proteins (*sxtM* and *sxtF*) and acyl-CoA N-acyltransferase (*sxtR*). The third class includes genes that are shared with Oscillatoriales cyanobacteria, specifically, *Lyngbya* sp., that belongs to the same genus of the STX+ *Lyngbya wollei* [14] and/or the bloom-forming and neurotoxin-producing *Trichodesmium erythraeum* [15]. This group is comprised of seven genes: sterole desaturase (*sxtD*), GDSL-lipase (*sxtL*), *O*-carbamoyltransferase (*sxtI*), sulfotransferase (*sxtN*), cephalosporin hydroxylase (*sxtX*), and two hypothetical genes (*sxtJ* and *sxtK*). The phylogeny of sxtN (Figure 3) provides an example of a STX+ gene of a non-Nostocaceae cyanobacterial provenance. *SxtN* is postulated to encode a sulfotransferase that carries out the transfer of the sulfate group from 3′-phosphoadenosine 5′-phosphosulfate (PAPS) to the carbamoyl group of saxitoxin compounds, i.e., based on work done with the STX+ dinoflagellate *Gymnodinium catenatum* [3]. However, unlike other STX genes, we could not identify a homolog of *sxtX* in ACBU02 (although it is encoded by three other STX+ cyanobacteria, *Cylindrospermopsis raciborskii* T3, *Lyngbya wollei* Carmichael/Alabama, and *Aphanizomenon flos-aquae* NH-5). This result is consistent with the prediction of Kellmann et al. [6], that the loss of *sxtX* from *Anabaena circinalis* (e.g., ACBU02) explains the inability of the toxic strains of this species to produce N-1-hydroxylated analogs of STX.

It is important to note that the different phylogenetic patterns observed in this group of genes could be explained by inter-phylum HGT, whereby the STX genes were horizontally transferred among specific cyanobacterial phyla, including the common ancestor of STX+ cyanobacterium. Alternatively, there may have been independent gene losses among cyanobacteria leading to the sporadic distribution of these sequences among the different lineages in our trees. One piece of evidence that favors the latter interpretation is the nested pattern of STX+ and STX− strains of *Anabaena circinalis* (Figure 1B). This complex pattern suggests the loss of saxitoxin production is a common phenomenon. Therefore the loss of the genes encoding this trait is also likely to be common among cyanobacteria, potentially explaining the phylogenetic distribution of some (or all) of these 13 putative STX genes.

### Genes of non-cyanobacterial origin

For the remaining 9 STX genes, we postulate they are of non-cyanobacterial origin. Interestingly, these can be clustered into 8 unique families, including enzymes that have been predicted to play key roles in the biosynthesis of saxitoxin (see Figure S1); e.g., the initial and unique Claisen-type condensation, carbamoylation, cyclization, and epoxidation [16]. These genes, which trace their origin to putative HGTs are polyketide synthase (*sxtA*, see below), cytidine deaminase (*sxtB*), amidinotransferase (*sxtG*), phenylpropionate dioxygenase (*sxtH* and *sxtT*), phytanoyl-CoA dioxygenase (*sxtS*), chaperone-like protein (*sxtE*), and two proteins of unknown functions (*sxtC* and *sxtQ*). Five of these 9 genes are of proteobacterial origin (e.g., sxtB; Table 1, group III).

Two genes (sxtH and sxtT) that encode phenylpropionate dioxygenase with a conserved Rieske domain show (Figure S1) a clear separation between STX+ and STX− cyanobacteria. Although the phylogeny indicates that the closest neighbor to the STX+ cyanobacterial clade is verrucomicrobial, there is an absence of bootstrap support for this relationship. Therefore we could not confidently designate a potential origin for these two genes.

One gene of unknown origin, *sxtC* (GenBank accession: ABI75092), with a postulated regulatory role in the biosynthesis of saxitoxin [6], shared significant identity with a single predicted protein in ACBU02. We performed a protein structure search using SMART [17] and predicted, albeit with low support, that sxtC belongs to the SCOP [18] superfamily of alpha/beta-hydrolases (SCOP accession: 53474). Then we searched for homologous proteins with permissive BLAST parameters (e.g., *e*-value<50) and using phylogenetic screening, we predicted that protein BH0493 from *Bacillus halodurans* is the closest relative to *sxtC*. BH0493 was recently characterized via the determination of its X-ray crystal structure [19] as an uronate isomerase, a member of the superfamily of amidohydrolases, catalyzing the isomerization of D-galacturonate to D-tagaturonate. Therefore, we hypothesize that the protein encoded by *sxtC* has an amidohydrolase activity with a role in the hydrolysis of the carbamoyl group of saxitoxin, resulting in decarbamoylated analogs (dcSTX), that have been shown to be produced by the two STX+ cyanobacteria *Anabaena circinalis* [20] and *Cylindrospermopsis raciborskii* [5].

**Figure 4:**
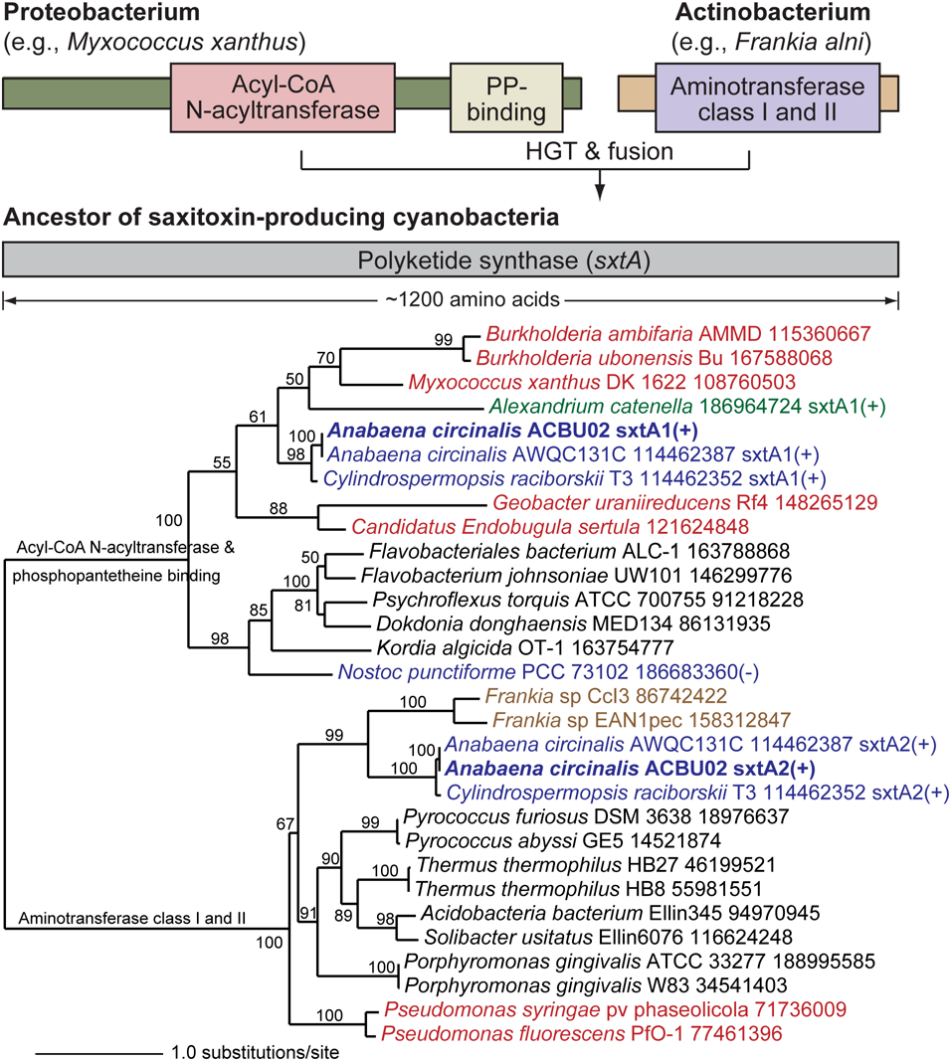
Gene fusion origin of sxtA. (a) Schematic diagram illustrating the fusion event that resulted in sxtA (polyketide synthase) in STX+ cyanobacteria. (b) ML phylogenetic tree of sxtA after dividing it into its two phylogenetic components; i.e., sxtA1 (a gene encoding acyl-CoA N-acyltransferase and phosphopantetheine binding domain-containing protein) and sxtA2 (a gene encoding aminotransferase class I and II). ML bootstrap values (when ≥50%) are indicated at the nodes. The branch lengths are proportional to the number of substitutions per site (see scale in the figure). Cyanobacterial taxa are shown in blue text, actinobacteria in brown text, proteobacteria in red text, and other non-cyanobacterial prokaryotes in black. The tree was rooted on the branch separating the two gene fusion partners.

Finally, the most intriguing member of the non-cyanobacterial group is sxtA that encodes a polyketide synthase. A BLAST search against the RefSeq protein database (Release 33) shows that sxtA is comprised of two evolutionarily distinct regions (Figure 4). The first region of ca. 800 amino acids is homologous to a polyketide synthase (GenBank accession: YP_632118) from taxa such as the deltaproteobacterium *Myxococcus xanthus* with two major regions specified by an acyl-CoA N-acyltransferase and a phosphopantetheine (PP) binding domain. The C-terminal ca. 390 amino acids however shares significant identity to a class I and II aminotransferase (GenBank accession: YP_482822) from actinobacteria such as *Frankia* sp. This structure suggests a chimeric origin of sxtA. ML tree inference verifies this hypothesis by demonstrating two significant, different phylogenetic signals that support the chimeric origin model (Figure 4). Therefore, we propose that the establishment of *sxtA* in the ancestor of STX-producing cyanobacteria occurred via two evolutionary phases. First, two independent HGT events, from the two different sources, contributed both regions to the genome of the ancestor of STX-producing cyanobacteria. Second, a fusion of the two genomic regions resulted in a *de novo* multidomain *sxtA* gene that is predicted to be the first enzyme in the biosynthesis of STX. Interestingly, a homolog of this key protein in STX synthesis was found in single-pass expressed sequence tag (EST) data from the STX+ dinoflagellate *Alexandrium catenella* [21] (Figure 4). This partial gene fragment provides the first evidence for the origin of STX genes in dinoflagellates *via* HGT.

### Phylogenomic analysis

**Figure 5:**
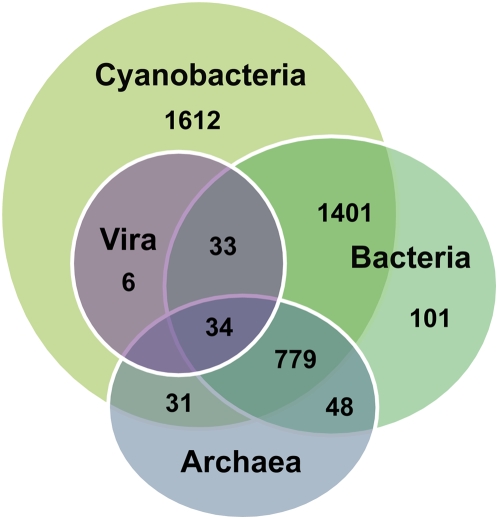
Origin of ACBU02 genes. Venn diagram showing the genome-wide distribution of genes in the STX+ cyanobacterium *Anabaena circinalis* ACBU02 with respect to sequences shared with other prokaryotes and viruses.

We carried out a genome-wide phylogenomic analysis to estimate the extent of HGT in the genome of ACBU02. This was done by determining the phylogenetic position of each of the predicted proteins in this genome followed by their clustering into major distinct phylogenetic patterns of origin (i.e., cyanobacterial, common to cyanobacteria and other bacteria, bacterial origin but not shared with cyanobacteria except ACBU02, archaeal, and viral). With bootstrap support ≥50%, we identified 1,612 genes (35% of the inferred trees) that are specific to cyanobacteria, 1,401 genes (30.5%) that are shared with other cyanobacteria with a bacterial origin, 779 genes (16.9%) that are common to cyanobacteria, bacteria, and archaea, and 73 genes (1.6%) with a viral origin. The Venn diagram shown in Figure 5 summarizes these results indicating that multiple evolutionary origins have contributed to the ACBU02 genome. This highly chimeric nature agrees with the nature of other cyanobacterial genomes [22].

In addition, we used genome-wide phylogenomics to compare the predicted genes in the two *Anabaena circinalis* strains ACBU02 (STX+) and ACFR02 (STX−) with other cyanobacteria and bacteria. We found that 95% of the genes were shared between the two strains and 14% of these shared genes were specific to ACBU02 and ACFR02. The remaining 5% of the genes were found only in ACBU02 and primarily shared with Nostocales (27%), Chroococcales (20%), Nostocales and Chroococcales (16%), and Oscillatoriales (4%), and represent potential gene losses in the STX− strain.

**Figure 6:**
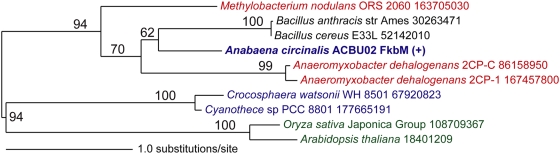
Phylogeny of SAM-dependent methyltransferase. Unrooted ML phylogenetic tree of a putative STX+ specific S-adenosyl-L-methionine (SAM)-dependent methyltransferase that has a potential *Bacillus*-like origin. ML bootstrap values (when ≥50%) are indicated at the nodes. The branch lengths are proportional to the number of substitutions per site (see scale in the figure). Cyanobacterial taxa are shown in blue text, non-cyanobacterial prokaryotes in black text, and eukaryotes (Plantae) in green.

Kellmann et al. [6] used iterated PSI-BLAST [23] that allows identification of homologs with low protein identity to suggest that sxtA contained a S-adenosyl-L-methionine (SAM)-dependent methyltransferase. However, in our phylogenomic analysis, we identified two SAM-dependent methyltransferases in ACBU02. The first belongs to the methyltransferase type 11 family and is shared between the two *Anabaena circinalis* strains and is ubiquitous among different cyanobacterial genera with a putative role in the of biosynthesis of ubiquinone. The second methyltransferase did not have a homolog in ACFR02 and the best BLAST hit (66% amino acid similarity and 40% identity) was a SAM-dependent methyltransferase FkbM from the firmicute *Bacillus cereus*. The closest cyanobacterial homolog was a methyltransferase FkbM from *Cyanothece* with 41% similarity and 22% identity. A structure-based search using PDBsum [24] supported the annotation as methyltransferase FkbM (PDB accession: 2py6). Our phylogenomic analysis predicted that the STX+ *Anabaena circinalis* SAM-dependent methyltransferase FkbM is specific to the STX+ strain and is not of cyanobacterial origin (Figure 6). Interestingly, of the two identified SAM-dependent methyltransferases, only FkbM has a significant similarity (*e*-value = 6.7E-5) to an EST from the STX+ dinoflagellate *Alexandrium tamarense*. We are currently exploring this second intriguing phylogenetic connection between STX biosynthesis genes in the toxic cyanobacterium and the toxic alga.

### Conclusions

Our results provide evidence that the assembly of putative STX genes in ACBU02 involved multiple HGT events from different sources, followed by coordination of the expression of foreign and native genes. The complexity of these steps could explain the limited distribution of this machinery among different, often closely related evolutionary lineages. Once established, loss of toxicity appears however to be widespread among closely related strains that have a STX+ ancestor. Despite this evolutionary complexity, saxitoxin is produced by members of two different kingdoms of life: cyanobacteria and dinoflagellates. This peculiar phylogenetic distribution leads to further questions about how and why the saxitoxin production pathway evolved in these two distantly related lineages. Did dinoflagellates gain the machinery via a single or multiple HGT events from STX+ cyanobacteria? Did the biosynthesis pathway evolve independently in the two lineages? Alternatively, do dinoflagellates host bacterial endosymbionts that produce or contribute key intermediates for STX production? In order to address these questions, additional genome data are needed from these different lineages, including closely related toxic and non-toxic strains from both prokaryotic and eukaryotic candidate species (work underway). These genome-scale investigations will provide insights into the possibility that other lineages may also potentially gain the ability to produce STX via *de novo* assembly of this pathway. Given the serious impacts of HABs on human health and fisheries, it is of high importance to gain more insights into the origin and spread of STX genes in both prokaryotic and eukaryotic systems.

## Materials and Methods

### Cultures

*Anabaena circinalis* strains ACUB02 and ACFR02 were supplied by CSIRO Collection of Living Microalgae as xenic cultures. Cyanobacteria were grown in MLA media [25] with continual light (approx. 60 μmol m-2 s-1 PAR) and agitation. Thus far it has been impossible to render these strains axenic; however, the total number of bacterial strains was successfully reduced by lysozyme treatment (following [26]) and pour plating in low-phosphate MLA media solidified with agarose. To reduce overall bacterial numbers prior to DNA extractions, the cells were washed several times with water followed by low speed (300 g) centrifugation. Preliminary experiments indicated that this washing procedure reduced the bacterial load to less than 0.02% of the original in the case of ACBU02 and to 0.7% of the original in the case of ACFR02. Genomic DNA was extracted from washed cells in mid/late-logarithmic phase using a CTAB method modified from [27] and [28]. Briefly, cell pellets were digested with 0.5 to 2% SDS followed by 0.1 to 0.5 mg ml-1 proteinase K. DNA was purified by incubation with high salt/CTAB buffer and organic extraction. DNA was precipitated using ethanol and 3 M sodium acetate, pH 5.2, digested with RNAse A and resuspended in TE. ACBU02 genomic DNA was amplified using the GenomiPhi DNA Amplification Kit (GE Healthcare) following manufacturer's instructions. ACFR02 genomic DNA was not amplified.

### Sequencing

Whole genome amplified DNA from *Anabaena circinalis* ACBU02 was supplied to 454 Life Sciences and the University of Arizona Genomics Institute and was sequenced using the 454 GS20 and 454 GS FLX systems. Assembled genomes (using the Newbler assembler) were provided by each facility. The Newbler assemblies of ACBU02 were combined and then were reassembled using SeqMan Pro (DNASTAR, Inc). High-molecular weight DNA from *Anabaena circinalis* ACFR02 was supplied to the University of Iowa DNA facility and was sequenced using the 454 GS FLX platform. A Newbler-assembled genome was supplied by the facility.

### 16S rRNA analysis

The 16S rRNA sequences were gathered from previously published data and sequences generated in this study. RNA secondary structure-based alignments were obtained using MAFFT [29]. Phylogenetic trees were inferred using RAxML [30] under the general time-reversible (GTR) model [31] of DNA substitution with a gamma distribution and 100 replicate bootstrap analyses.

### STX protein trees

For the STX phylogenetic analyses, we started with a similarity search using BLAST that allowed up to 10,000 hits to be returned for each query, matching a significance threshold of *e*-value<1E-5. Next, we scanned the significant hits for each query and sampled the top hits from each taxonomic group, based on the NCBI taxonomic convention (see below for details). This approach ensured adequate taxon sampling for each query sequence without generating trees of unmanageable complexity. Thereafter, multiple sequence alignments (MSAs) were obtained using MAFFT [29], MSAs were refined manually, and phylogenies were inferred using RAxML [30] under the WAG model of evolution [32] with gamma distribution (WAG + Γ model) and 100 replicate bootstrap analysis.

### Genome annotation

The Rapid Annotation using Subsytems Technology (RAST) [33] was used to perform general genome annotation. Prediction of components that were missing from RAST subsystems were identified with the Hidden Markov Model (HMM)-based self-training system, GeneMarkS [11] for protein-coding genes and RNAmmer [34] for ribosomal RNA (rRNA) genes.

### Gene families

We performed a self-BLAST search for each predicted protein against the entire set of predicted proteins with *e*-value<1E-6. Afterwards, we used the collection of matching hits to guide the cluster of the gene into gene families using EasyCluster [35].

### Phylogenomic analysis

We constructed a database with more than 500 genomes that were obtained primarily from RefSeq [36], Joint Genome Institute (JGI) and EST libraries; e.g., TBestDB [37]. The database included the entire set of completely sequenced bacterial genomes (including cyanobacteria) and sampling of the following taxonomic groups, Amoebozoa, Animalia, Archaea, Chromalveolata, Excavata, Fungi, Plantae, and Rhizaria. Additionally, the database was augmented with more than 2,000 viral genomes, obtained from NCBI [38]. The complete set of the 5,188 predicted proteins were queried for homologs against the constructed database using WU-BLAST [39] with an adaptive two-point *e*-value threshold, 1E-5 and 1E-10 for non-conserved and conserved queries, respectively. In order to maximize the taxonomic diversity among the homologs for each query, while maintaining a reasonable number of homologs for downstream steps in the analysis, a maximum of five species was set for each bacterial phylum (i.e., Acidobacteria, Actinobacteria, Aquificae, Bacteroidales, Chlamydiae, Chlorobi, Chloroflexi, Deinococci, Firmicutes, Flavobacteria, Fusobacteria, Lentisphaerae, Planctomycetacia, Proteobacteria, Sphingobacteria, Spirochaetes, Synergistetes, Thermotogae and Verrucomicrobia). Then phylogenies were inferred as described in the phylogenetic analyses (above) except alignments were refined using Gblocks [40]. Finally, using PhyloSort [41], ML trees were grouped into different phylogenetic categories by matching topological criteria with at least 50% bootstrap support values, the topological criteria were defined to examine the ubiquity or specificity of each of the toxic *Anabaena circinalis* predicted genes among the different taxonomic groups with a focus on the detection of potential HGT scenarios among prokaryotic phyla and between the prokaryotic and viral kingdoms.

## Supporting Information

Figure S1 Trees of all STX proteins identified in Cylindrospermopsis raciborskii T3 The maximum likelihood bootstrap values are indicated at the nodes in red text. The branch lengths are proportional to the number of substitutions per site (see scale in the figure). The trees have been rooted arbitrarily. STX (+) strains are shown in blue text and the novel ACBU02 and ACFR02 sequences are shown in bold blue text.
(1.69 MB PDF)
