# Supplementary material for: Origin of Saxitoxin Biosynthetic Genes in Cyanobacteria
Source: PLoS One. 2009 Jun 1;4(6):e5758. doi: 10.1371/journal.pone.0005758 (PMC2684587; doi:10.1371/journal.pone.0005758)

sxtA: polyketide synthase

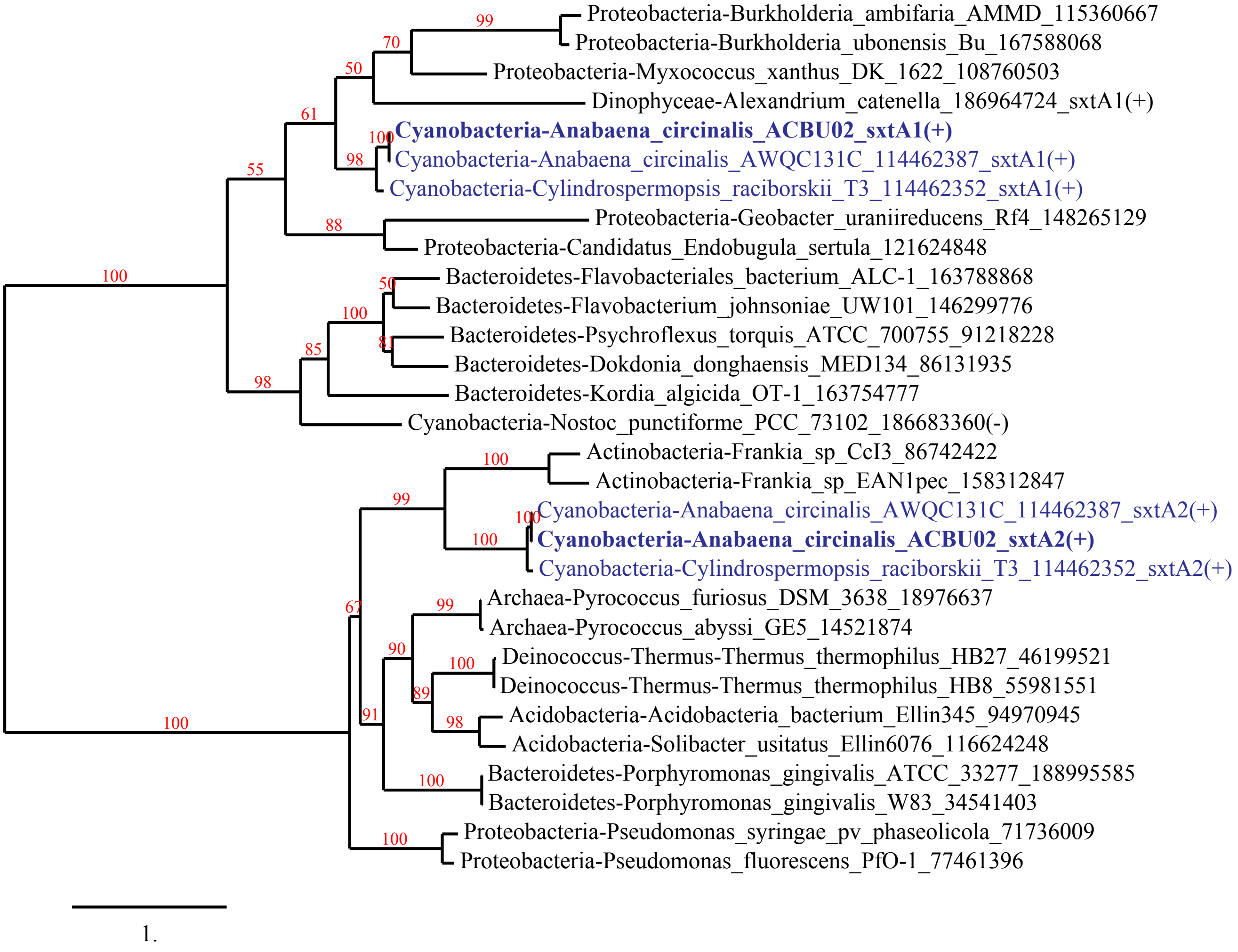

sxtB: Cytidine deaminase

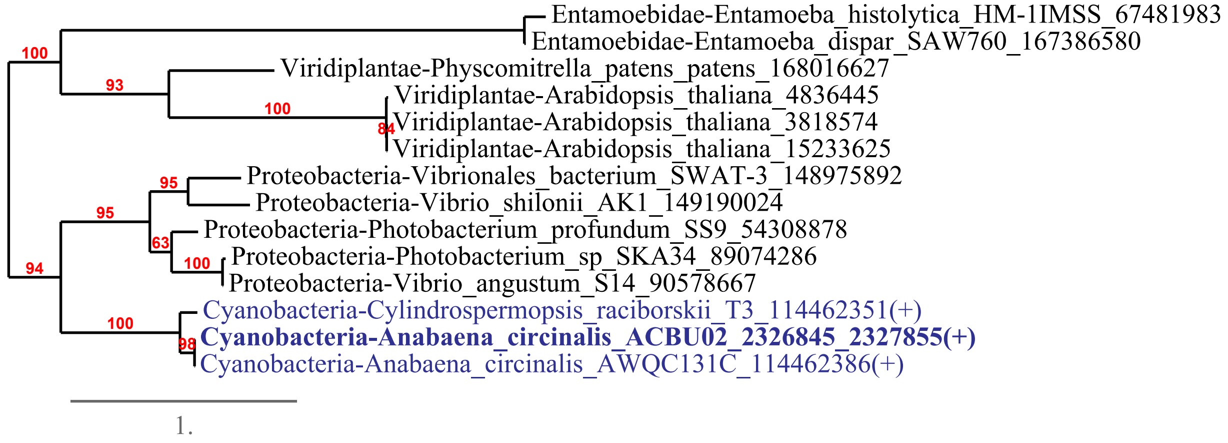

sxtC: Amidohydrolase

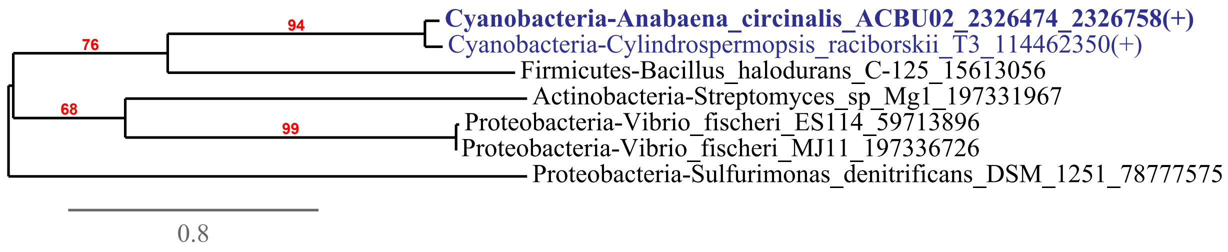

sxtD: sterole desaturase

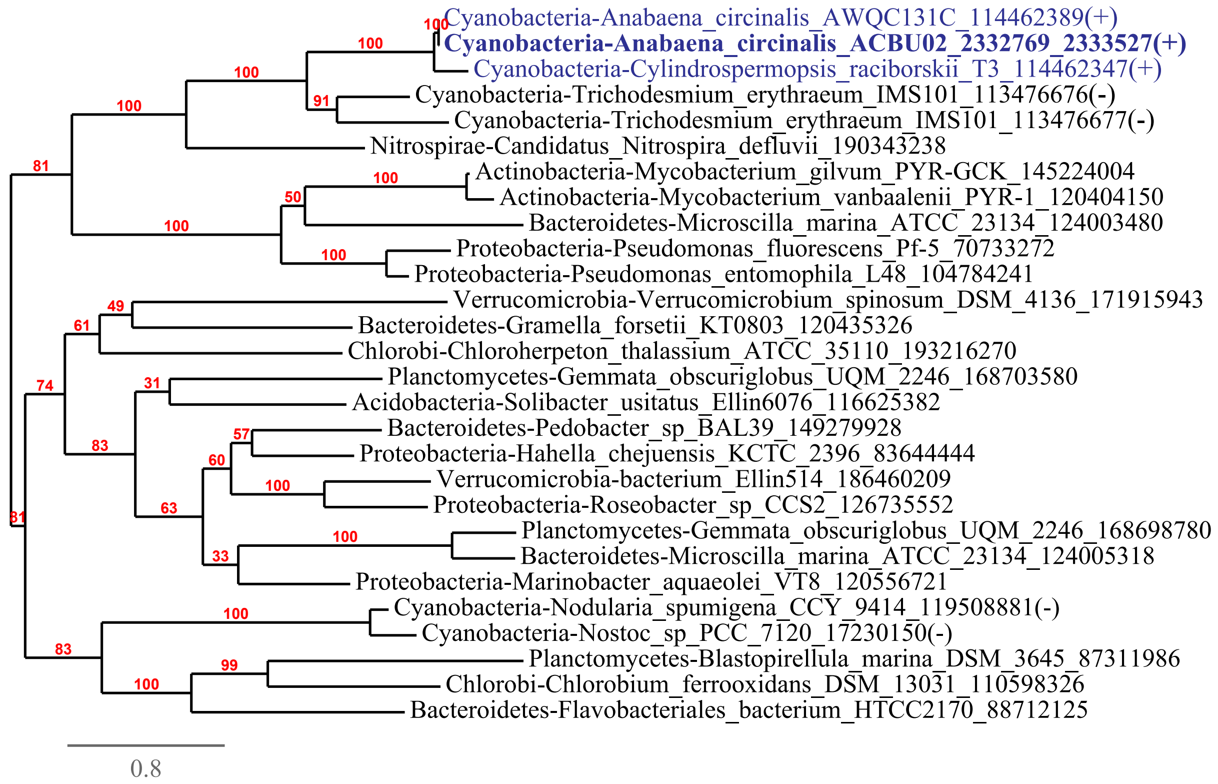

sxtE: Chaperone-like protein

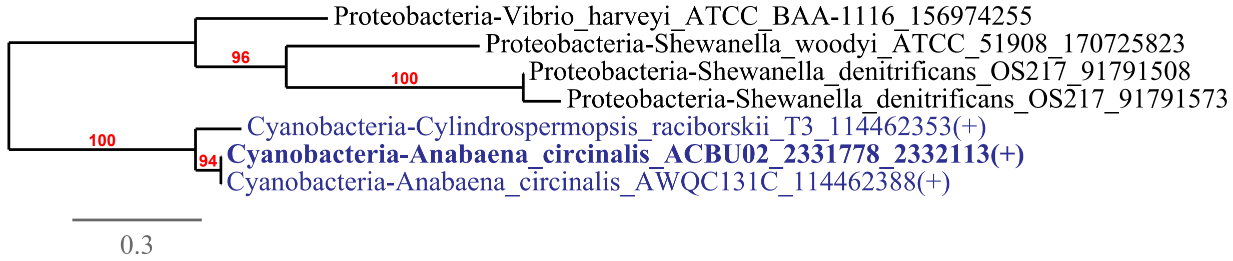

sxtF, sxtM: sodium-driven multidrug and toxic compound extrusion protein

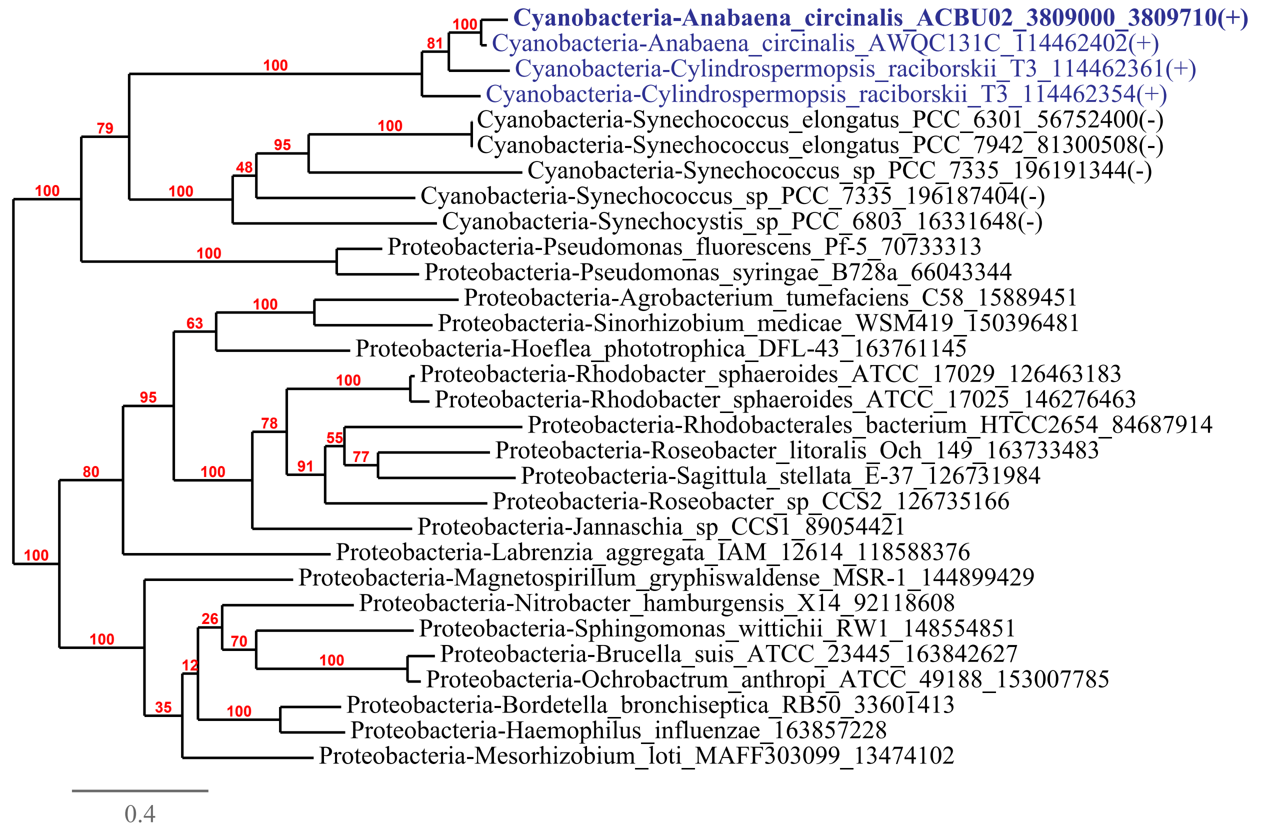

sxtG: Amidinotransferase

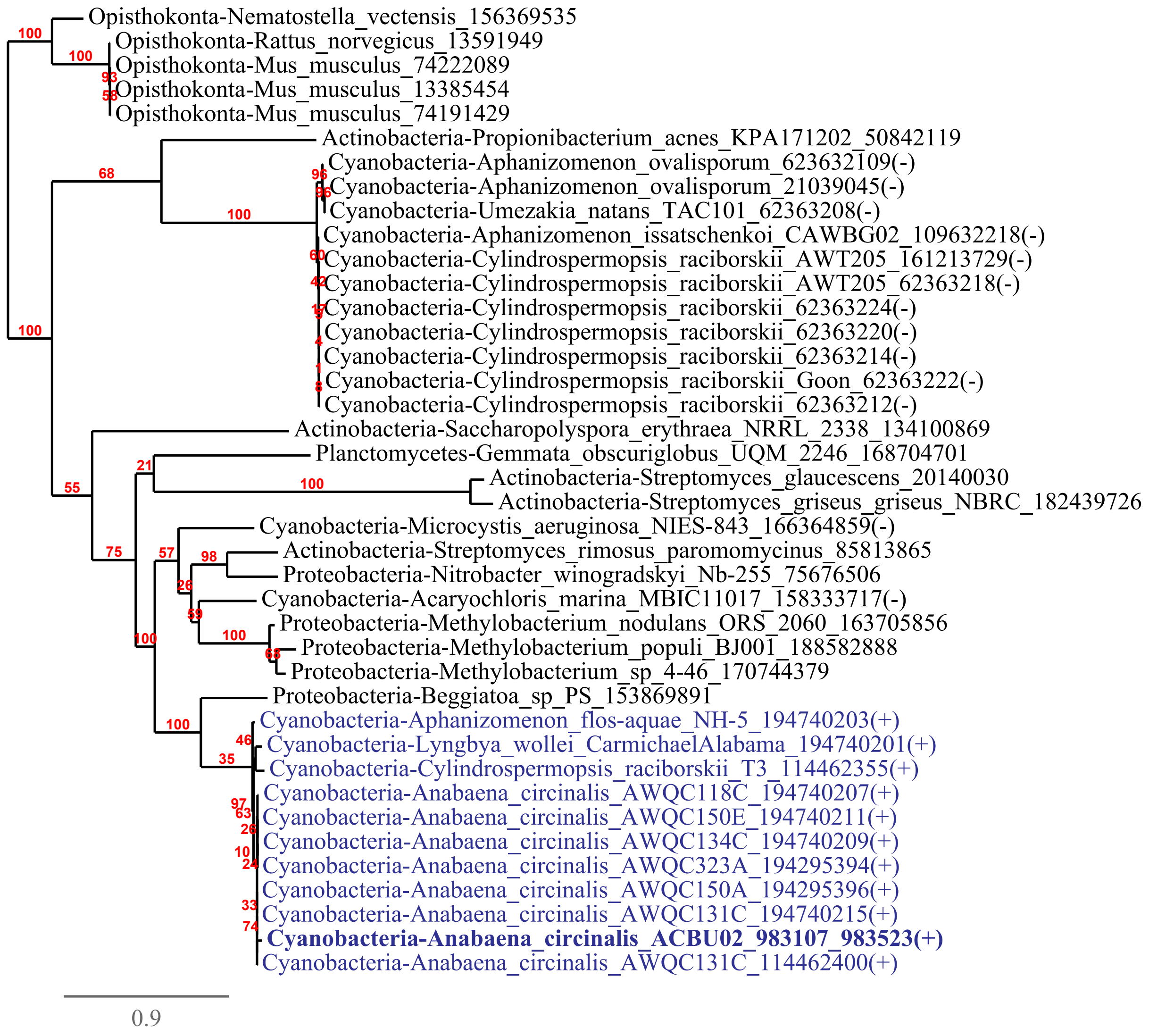

sxtH, sxtT: Phenylpropionate dioxygenase

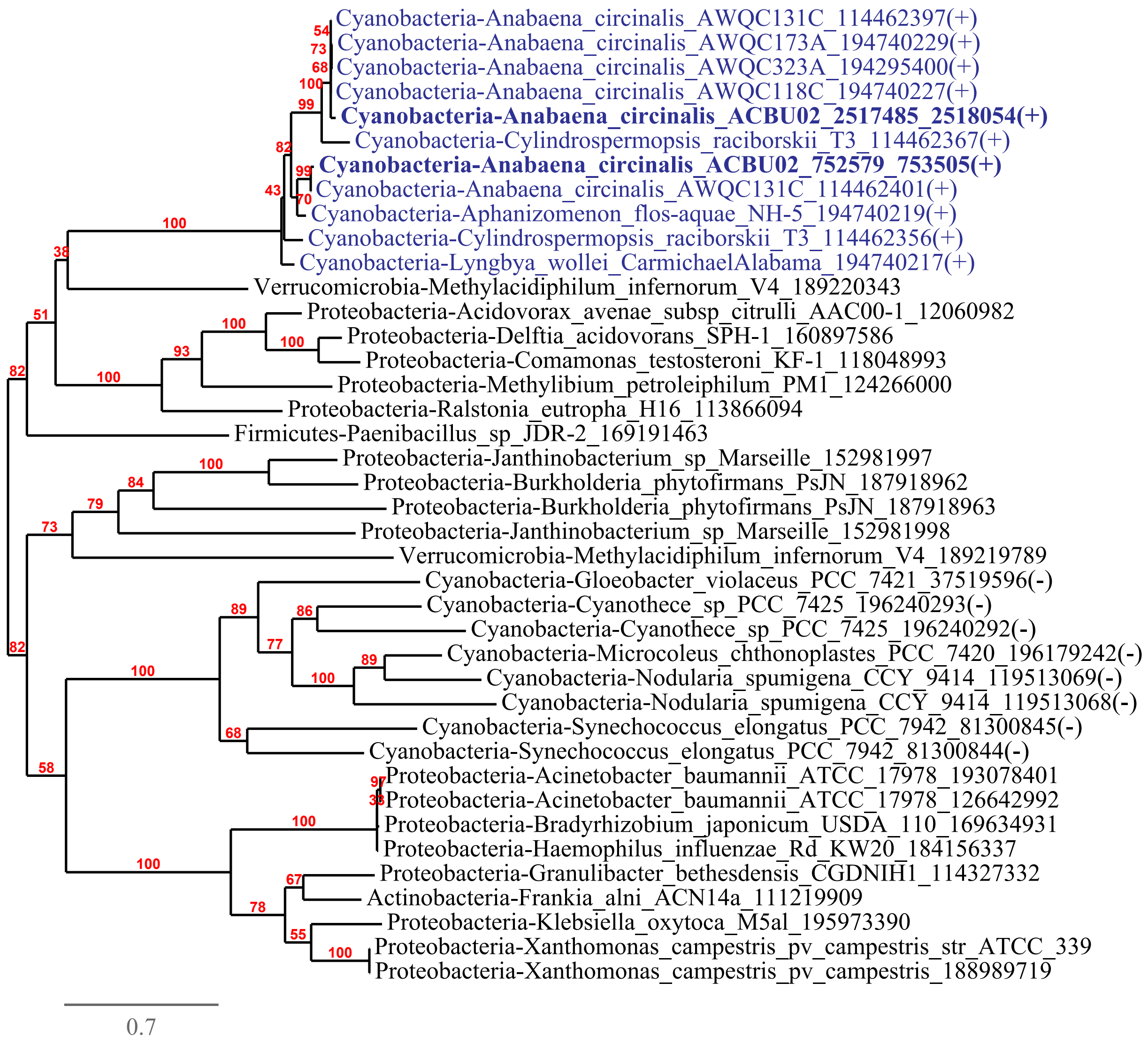

sxtI: *O*-carbamoyltransferase

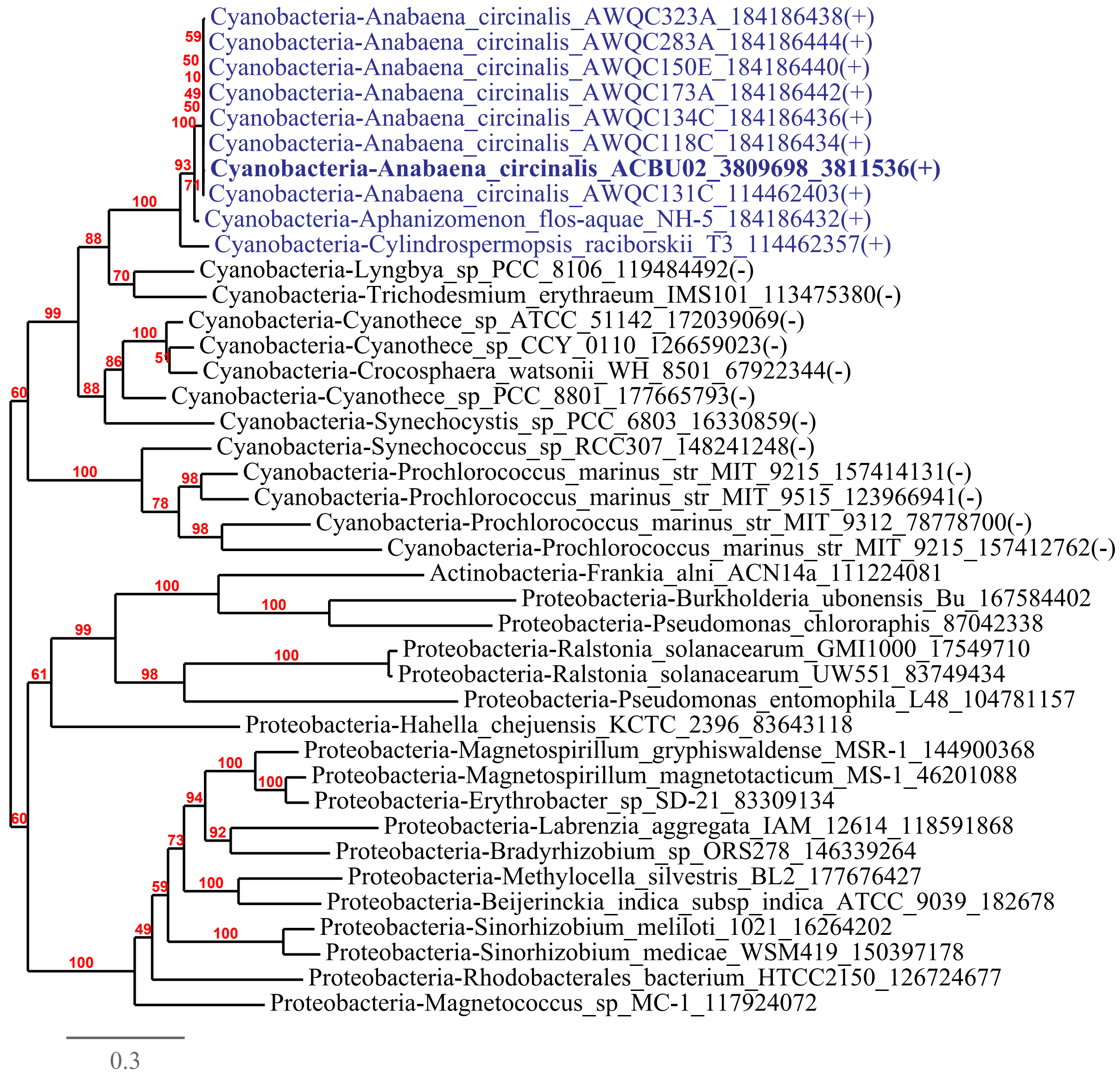

sxtJ: Hypothetical protein

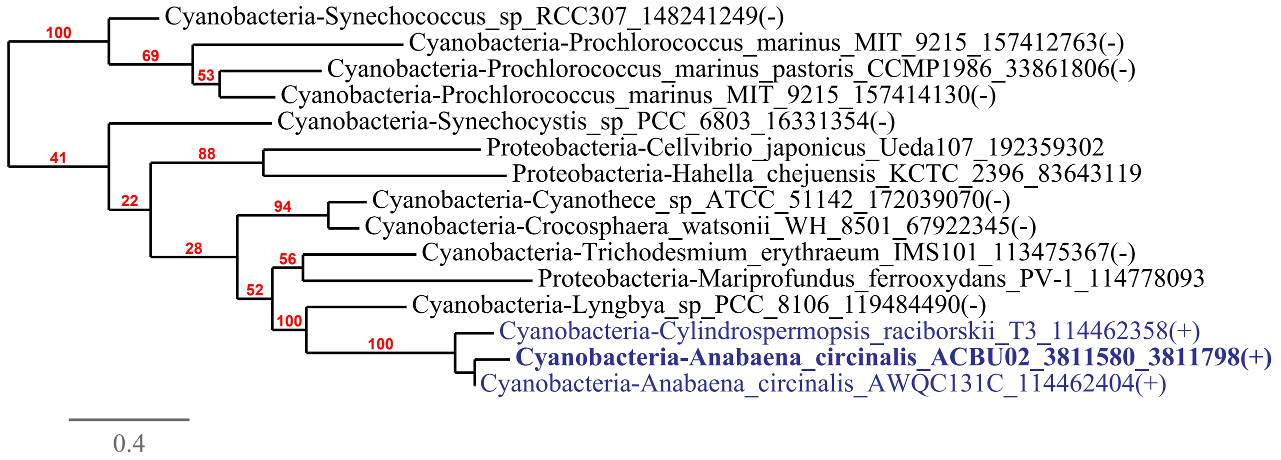

sxtK: Hypothetical protein

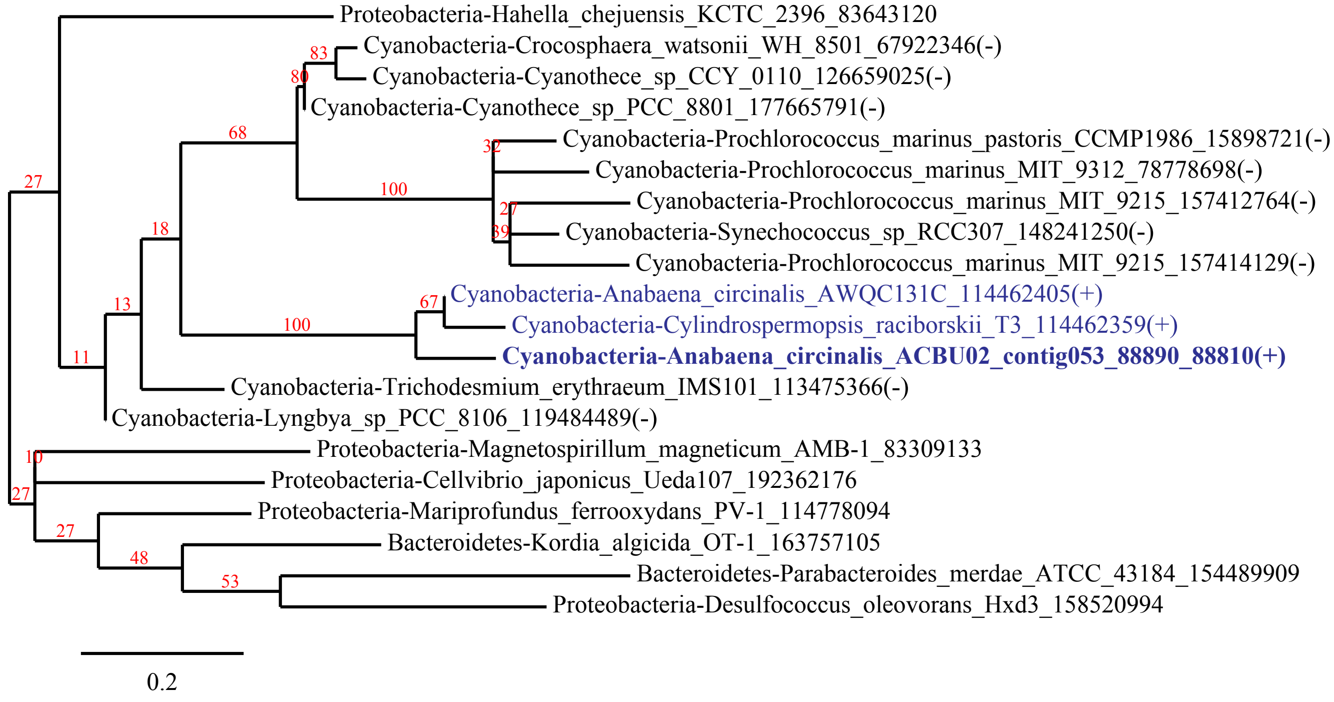

sxtL: GDSL-lipase

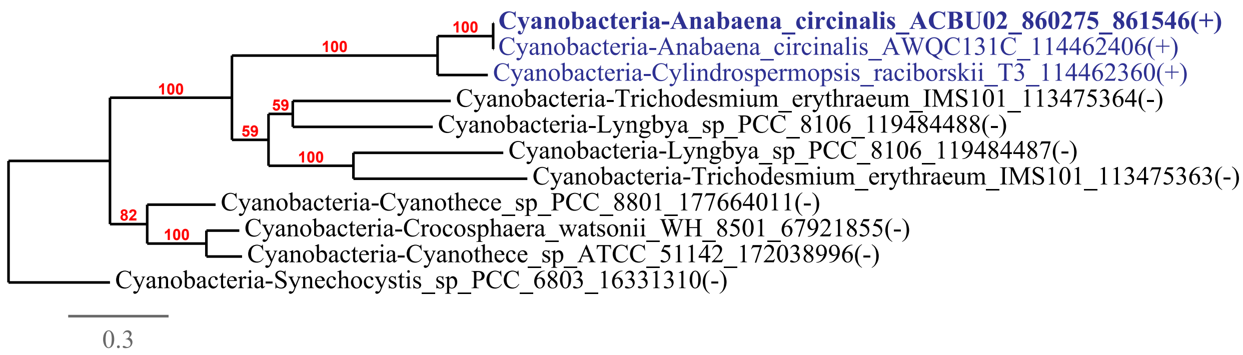

sxtN: Sulfotransferase

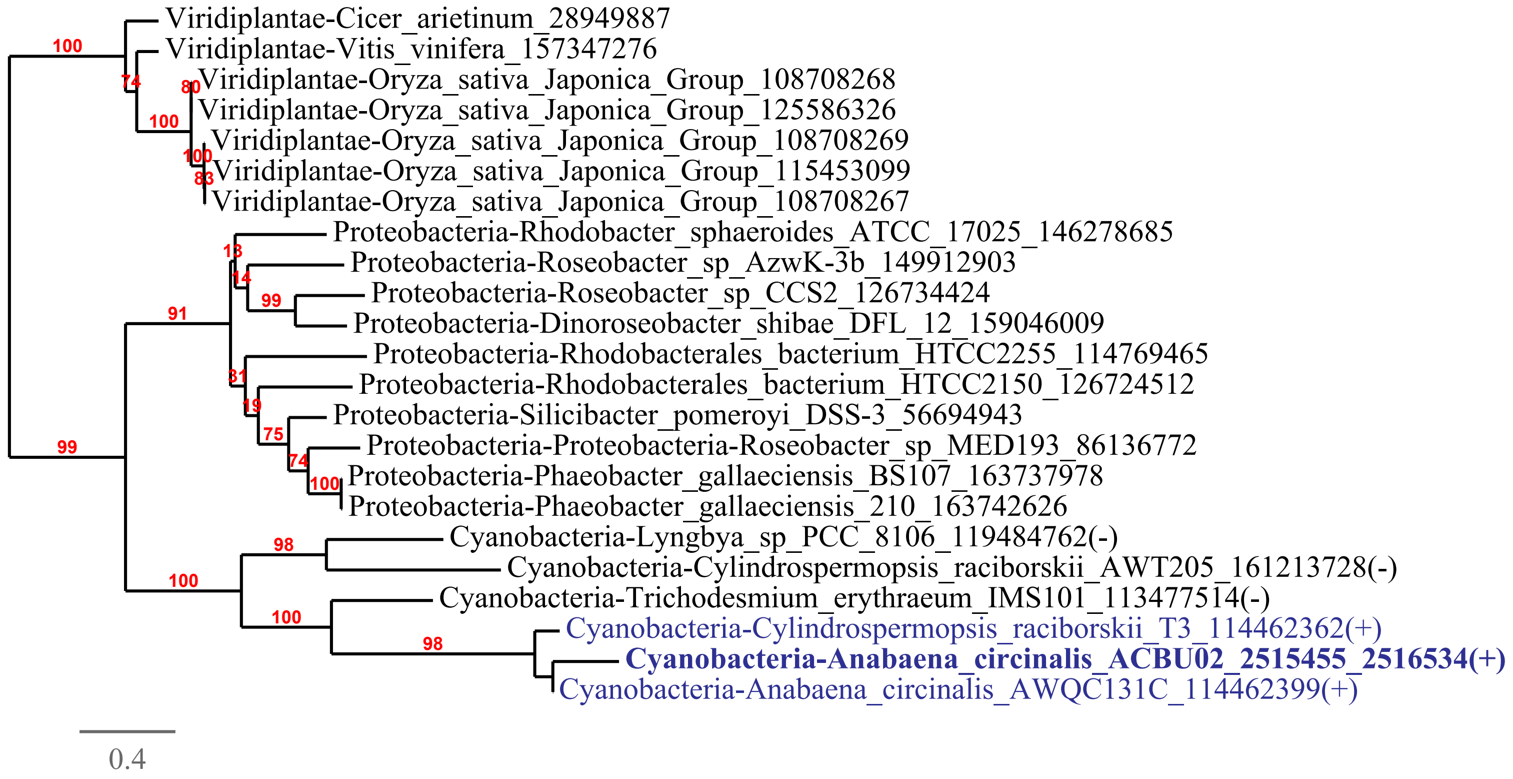

sxtO: Adenylylsulfate kinase

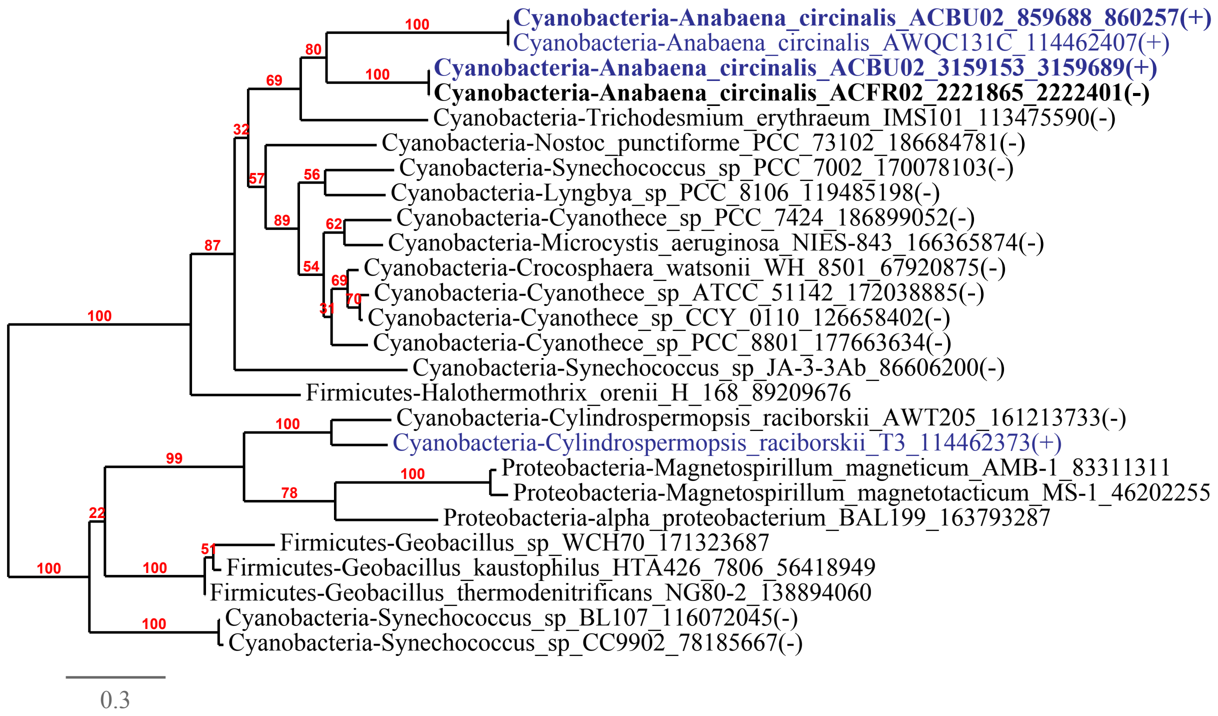

sxtP: Saxitoxin-binding protein

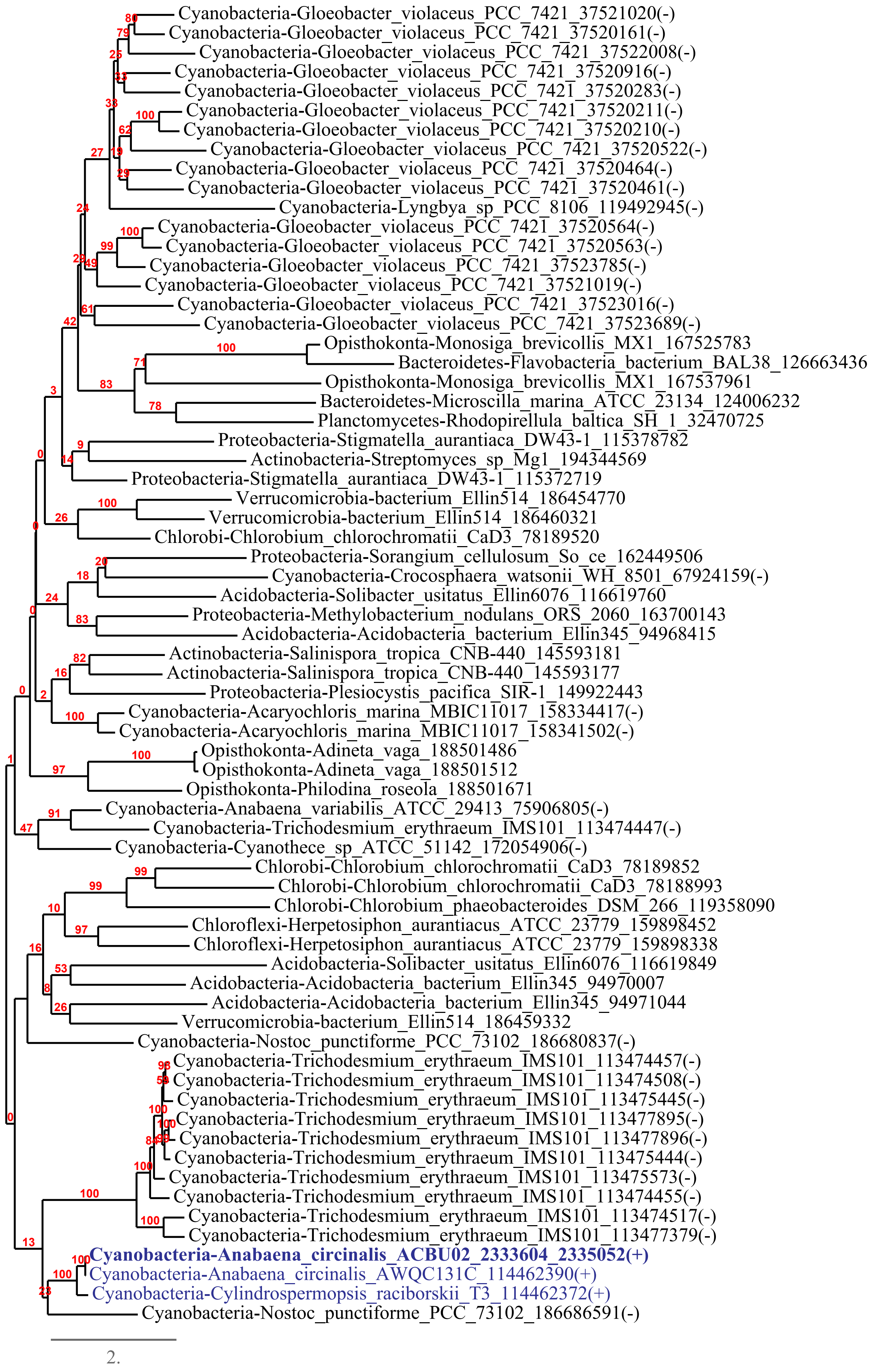

sxtQ: Unknown

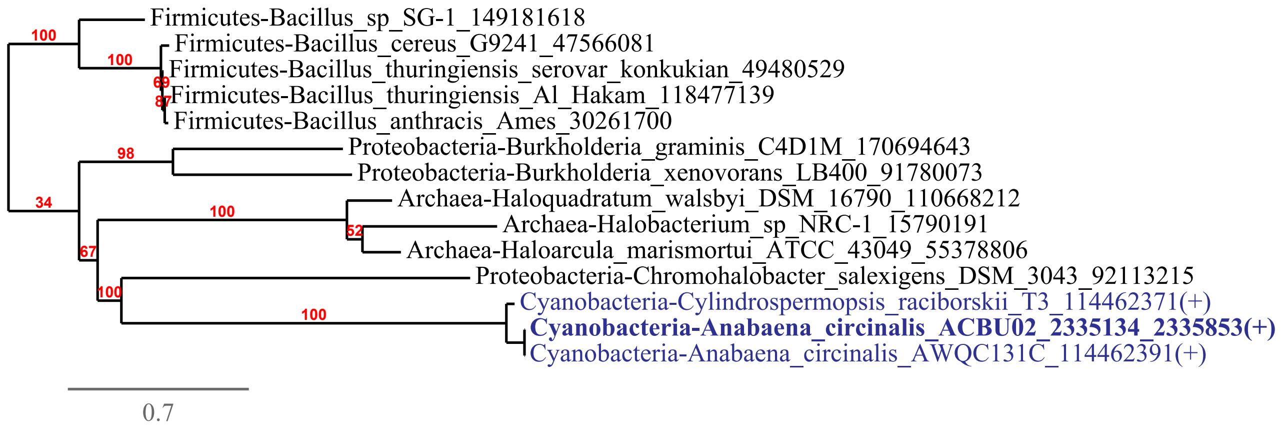

sxtR: Acyl-CoA N-acyltransferase

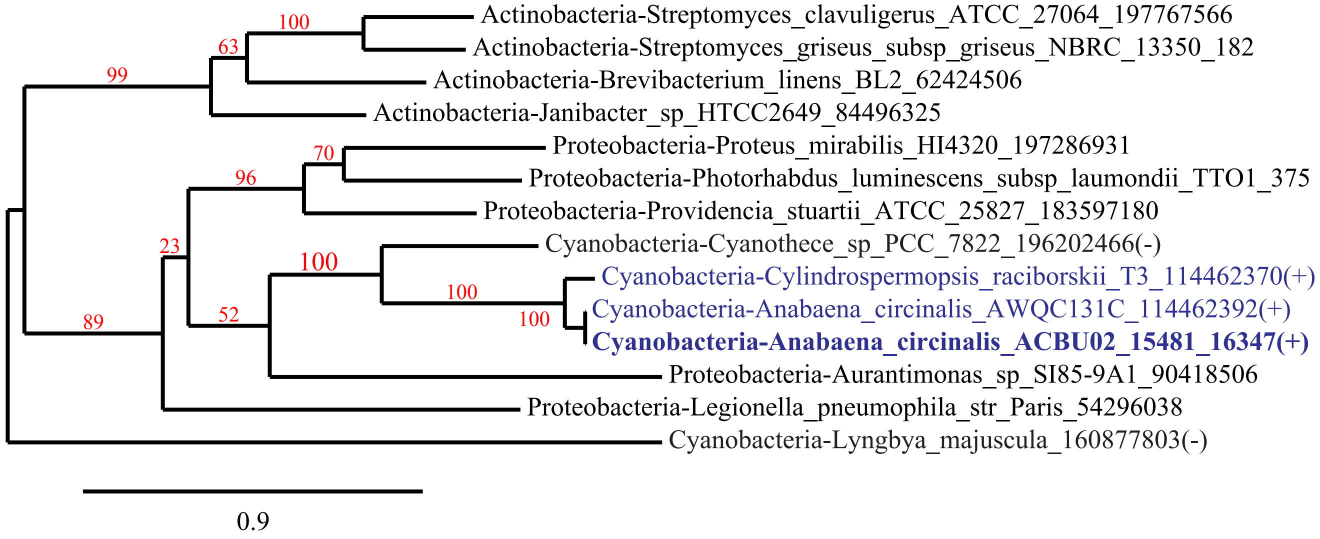

sxtS: Phytanoyl-CoA dioxygenase

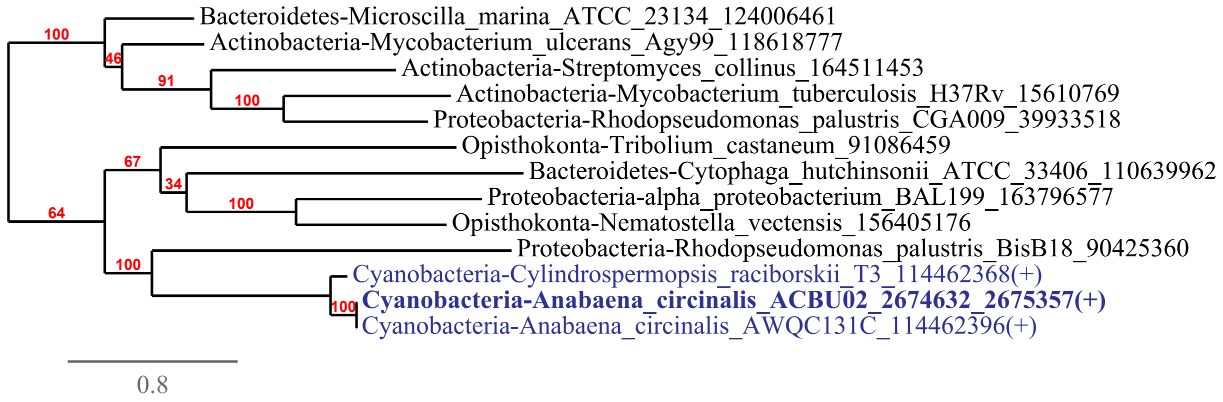

sxtU: Short-chain alcohol dehydrogenase

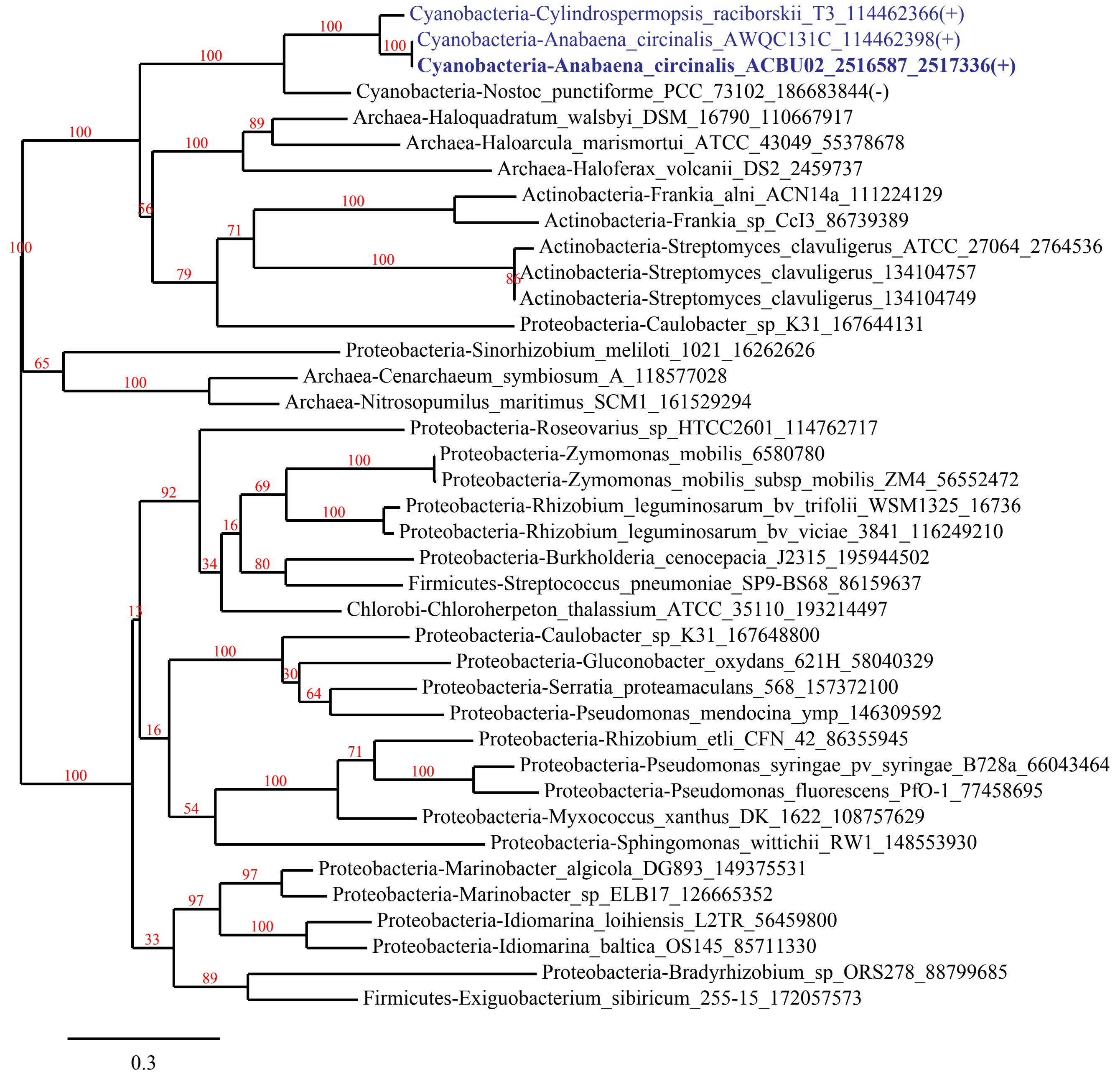

sxtV: succinate dehydrogenase/fumarate reductase

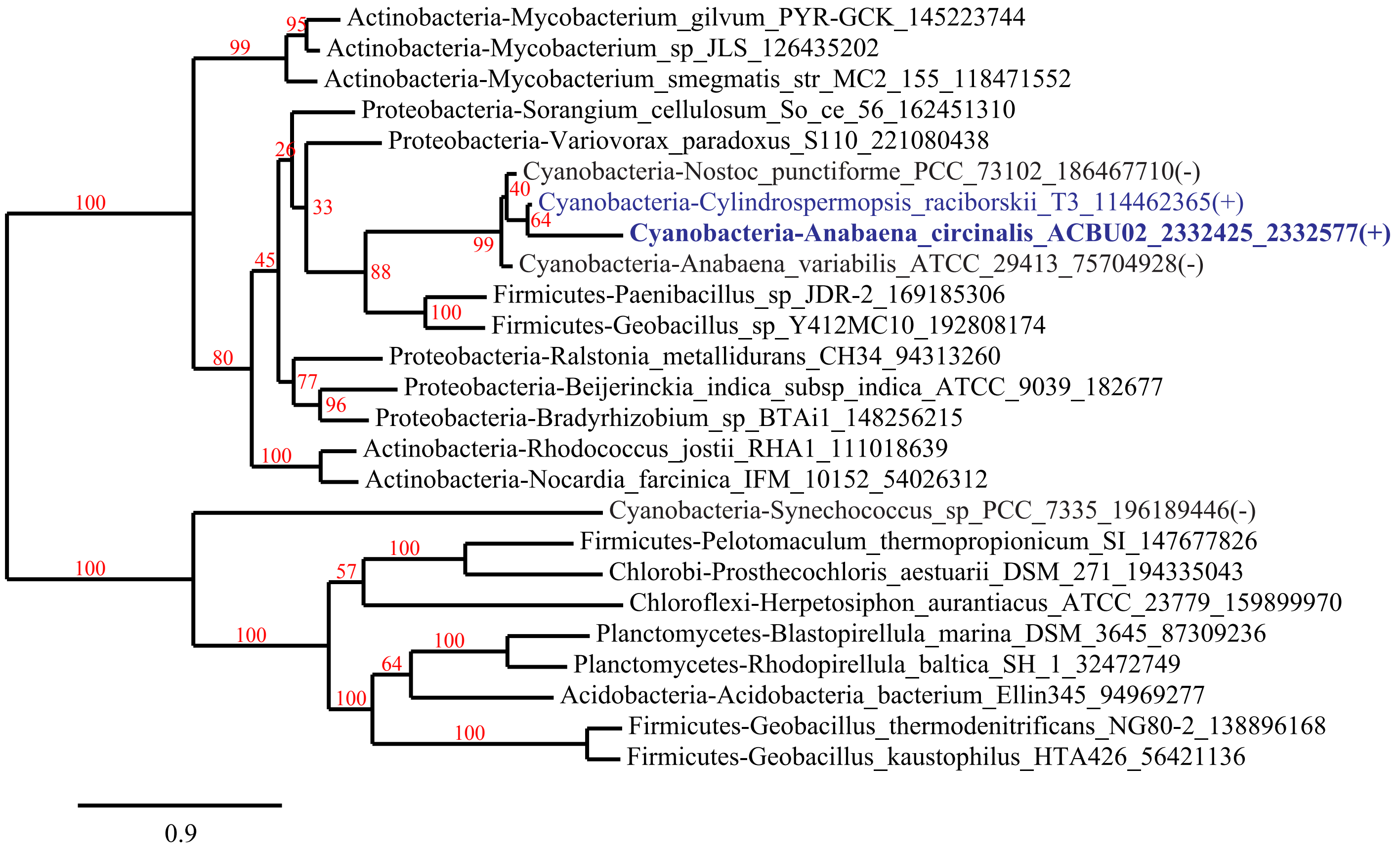

sxtW: ferredoxin

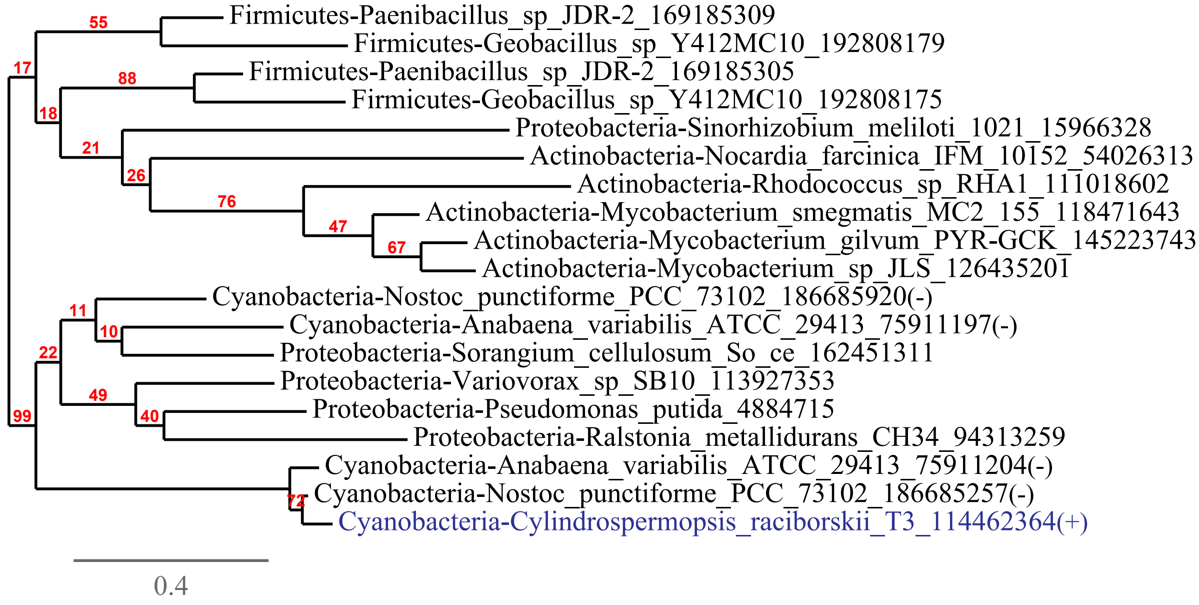

sxtX: Cephalosporin hydroxylase

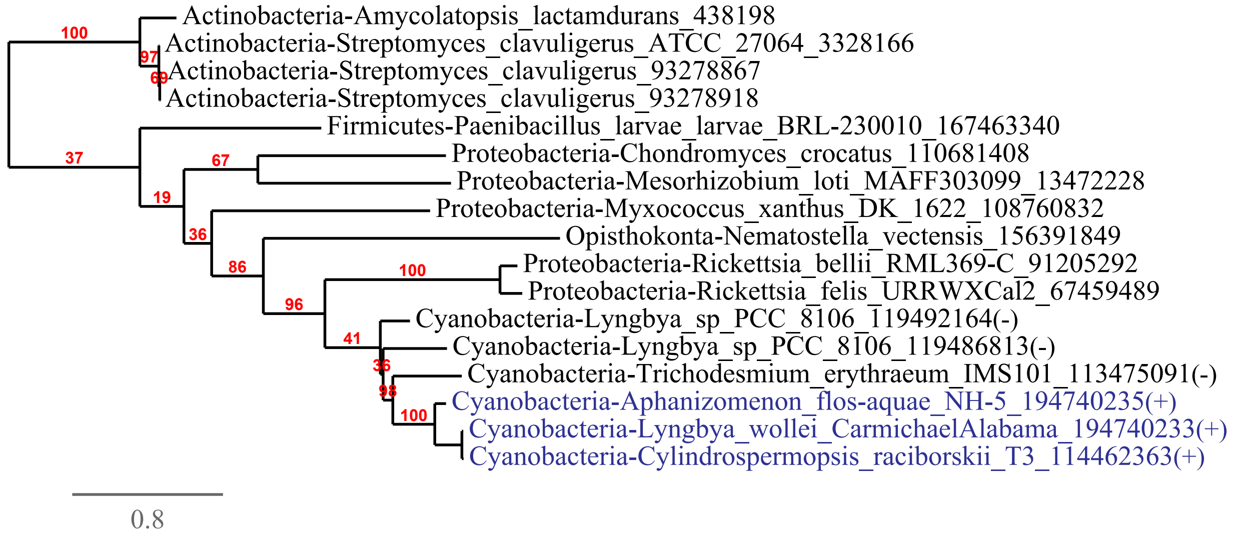

sxtY: Phosphate uptake regulator

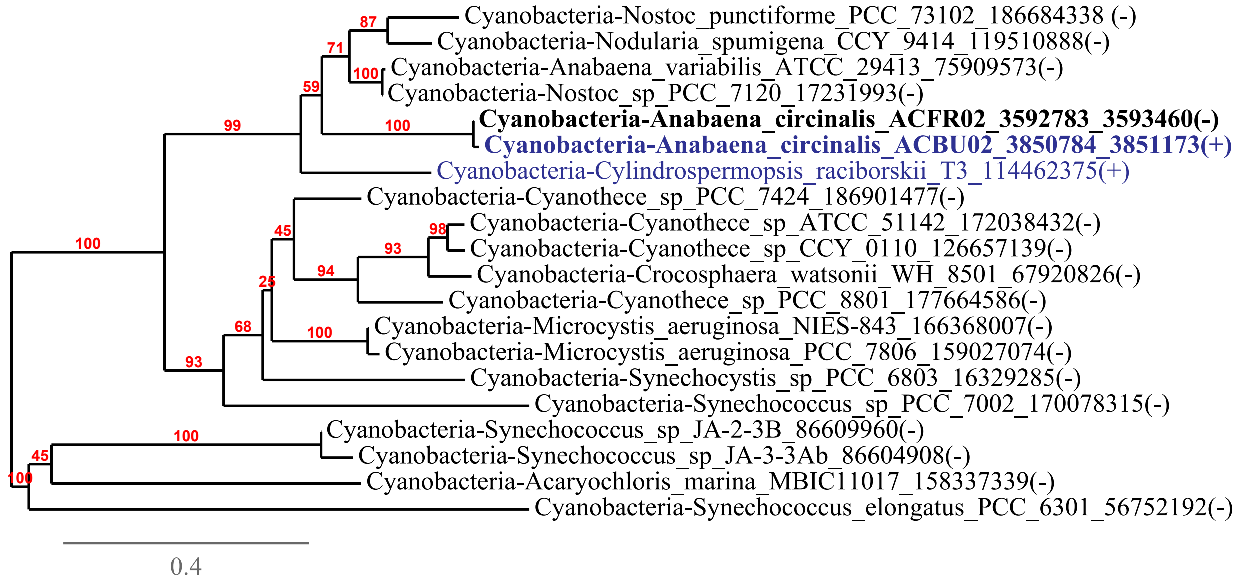

sxtZ: Two-component sensor histidine kinase

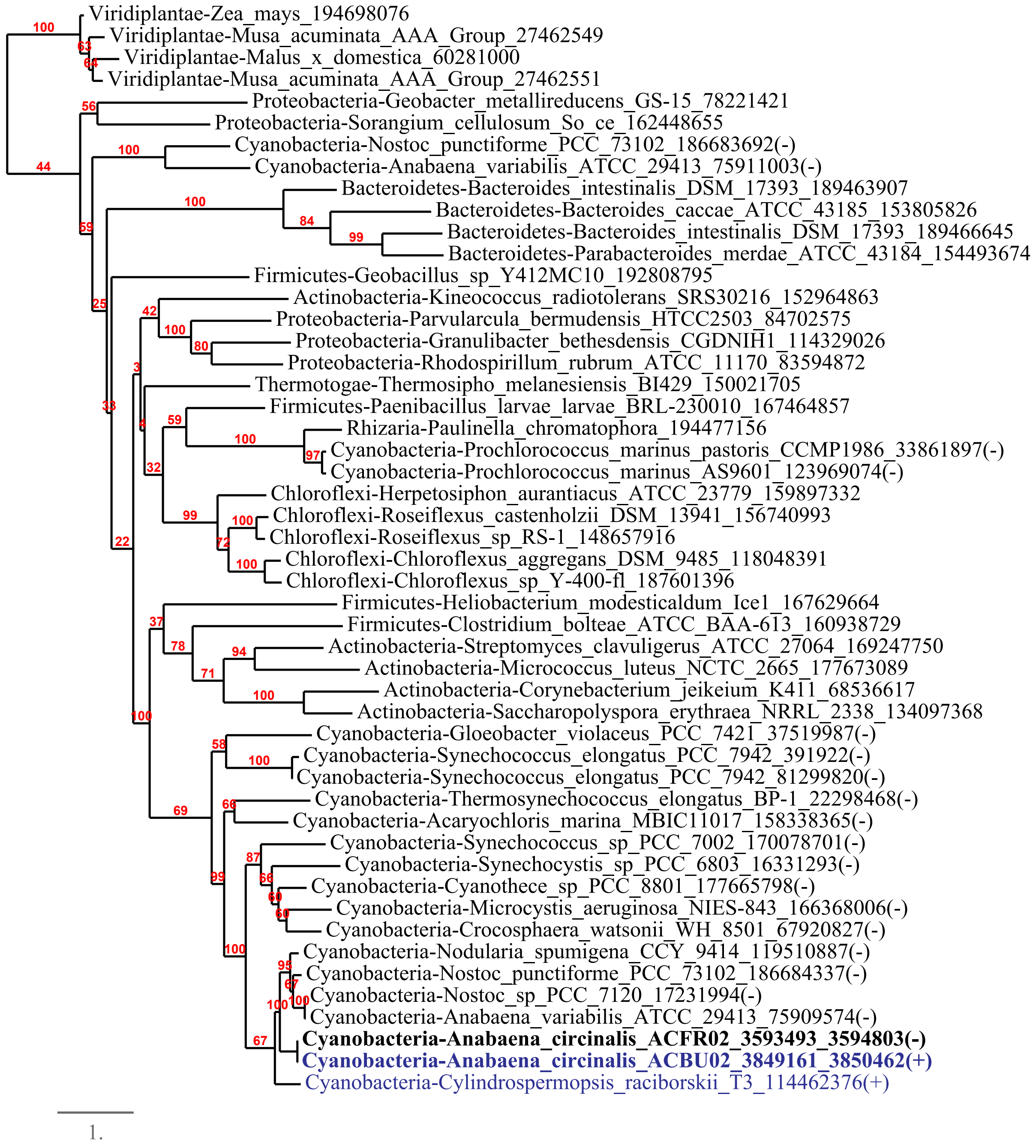

Supplement: Figure S1 — Trees of all STX proteins identified in Cylindrospermopsis raciborskii T3 The maximum likelihood bootstrap values are indicated at the nodes in red text. The branch lengths are proportional to the number of substitutions per site (see scale in the figure). The trees have been rooted arbitrarily. STX (+) strains are shown in blue text and the novel ACBU02 and ACFR02 sequences are shown in bold blue text. (1.69 MB PDF) [file pone.0005758.s001.pdf]
